# PAN-INTACT enables direct isolation of lineage-specific nuclei from fibrous tissues

**DOI:** 10.1371/journal.pone.0214677

**Published:** 2019-04-02

**Authors:** Samadrita Bhattacharyya, Adwait A. Sathe, Minoti Bhakta, Chao Xing, Nikhil V. Munshi

**Affiliations:** 1 Department of Internal Medicine, Division of Cardiology, UT Southwestern Medical Center, Dallas, Texas, United States of America; 2 McDermott Center for Human Growth and Development, UT Southwestern Medical Center, Dallas, Texas, United States of America; 3 Department of Molecular Biology, UT Southwestern Medical Center, Dallas, Texas, United States of America; 4 Hamon Center for Regenerative Science and Medicine, Dallas, Texas, United States of America; IRCCS San Raffaele Pisana, ITALY

## Abstract

Recent studies have highlighted the extraordinary cell type diversity that exists within mammalian organs, yet the molecular drivers of such heterogeneity remain elusive. To address this issue, much attention has been focused on profiling the transcriptome and epigenome of individual cell types. However, standard cell type isolation methods based on surface or fluorescent markers remain problematic for cells residing within organs with significant connective tissue. Since the nucleus contains both genomic and transcriptomic information, the isolation of nuclei tagged in specific cell types (INTACT) method provides an attractive solution. Although INTACT has been successfully applied to plants, flies, zebrafish, frogs, and mouse brain and adipose tissue, broad use across mammalian organs remains challenging. Here we describe the PAN-INTACT method, which can be used to isolate cell type specific nuclei from fibrous mouse organs, which are particularly problematic. As a proof-of-concept, we demonstrate successful isolation of cell type-specific nuclei from the mouse heart, which contains substantial connective tissue and harbors multiple cell types, including cardiomyocytes, fibroblasts, endothelial cells, and epicardial cells. Compared to established techniques, PAN-INTACT allows more rapid isolation of cardiac nuclei to facilitate downstream applications. We show cell type-specific isolation of nuclei from the hearts of Nkx2-5^Cre/+^; R26^Sun1-2xsf-GFP-6xmyc/+^ mice, which we confirm by expression of lineage markers. Furthermore, we perform Assay for Transposase Accessible Chromatin (ATAC)-Seq to provide high-fidelity chromatin accessibility maps of Nkx2-5^+^ nuclei. To extend the applicability of PAN-INTACT, we also demonstrate successful isolation of Wt1^+^ podocytes from adult kidney. Taken together, our data suggest that PAN-INTACT is broadly applicable for profiling the transcriptional and epigenetic landscape of specific cell types. Thus, we envision that our method can be used to systematically probe mechanistic details of cell type-specific functions within individual organs of intact mice.

## Introduction

Multicellular organisms harbor a single genome, yet exhibit a high degree of cell-type diversity specified by deployment of unique transcriptional networks. Identifying the mechanistic basis for such exquisite cell type specification is a fundamental question in biology and will help illuminate disease pathogenesis. In order to address these questions, many investigators study specific cell-types using artificial systems, such as cell lines [1, 2] and *in vitro* stem cell differentiation [3, 4]. Alternatively, individual regions within a native organ can be studied using laser capture microdissection (LCM) [5, 6], although specific cell types cannot be examined using LCM. In contrast, fluorescence-activated cell sorting (FACS) does allow purification of specific, fluorescently tagged cells or nuclei [7–10]. However, FACS is particularly suited for cells in suspension (e.g. cells obtained from whole blood) or soft tissues that are easily digested (e.g. brain and fetal heart). In contrast, many fibrous tissues and most adult organs require harsh digestion conditions to generate a single-cell suspension, which can adversely affect the resulting transcriptional and/or epigenetic profiles.

To overcome the need for harsh cellular digestion, a number of genetic methods have been developed. The Bacterial Artificial Chromosome (BAC) transgenic translating ribosome affinity purification (BAC-TRAP) approach is one popular tool for obtaining gene-expression signatures of individual cell-types within a tissue [11–13]. Although BAC-TRAP provides transcriptional profiles at high-resolution and the potential to obtain ribosome footprints, this technique cannot provide any genomic information. Alternatively, the bio Chromatin Immuno-Precipitation (ChIP)-seq method carries great potential for establishing p300 ChIP-Seq profiles in a cell type specific manner, but it remains limited by the availability of specific bio-tagged alleles and does not provide transcriptional information [14]. In contrast, the INTACT (Isolation of Nuclei TAgged in specific Cell Types) method [15–18] uses affinity purification of tagged nuclei to provide both genomic and transcriptomic information. INTACT was originally developed in plants [15, 16], but it has been rapidly adapted in worms, flies, fish, frogs, and mice [17–20]. In a recent study, a nuclear tag was knocked into the Rosa26 locus, and this mouse line was crossed with specific Cre driver lines to isolate rare neuronal subtypes for transcriptional and epigenetic profiling [17].

In addition to the mammalian brain, many other organ systems harbor a diverse array of unique cell types. For example, recent single-cell atlas data has highlighted the cell type heterogeneity within the embryonic heart in mice [21, 22]. Nevertheless, little is known about the mechanisms underlying such cell-type diversity. To profile bulk cardiomyocytes (CMs) from fibrous adult hearts, mouse and human CM nuclei have been isolated by FACS and magnetic-assisted sorting based on affinity purification using a Pericentriolar Material 1 (PCM1) antibody [9,10, 23–26]. Consequently, transcriptomic and epigenomic analyses of adult CMs from normal and diseased hearts have been described [10, 25–26]. However, existing methodologies cannot isolate lineage-specific nuclei from fibrous adult heart tissue for downstream transcriptional and epigenetic analysis.

Here, we present a highly versatile method (PAN-INTACT) that allows facile isolation of cell-type specific nuclei for downstream analysis from fibrous, problematic tissues such as heart and kidney. First, we establish and optimize parameters for extracting intact nuclei from adult heart tissue at various ages. Then, we provide sucrose gradient conditions that can be tuned to either preserve cell-type heterogeneity or isolate bulk CM nuclei without an antibody or genetic manipulation. Importantly, we establish and rigorously optimize a version of PAN-INTACT for use in mouse heart tissue, which we validate using parallel immunoaffinity purification of CM nuclei. Furthermore, we demonstrate that lineage-specific cardiac nuclei purified by PAN-INTACT can be used to generate high-quality chromatin accessibility maps. Finally, we display the versatility of PAN-INTACT by its application to a cell type in adult kidney—podocytes. Taken together, our study describes a sensitive, robust, and straightforward method for cell type specific transcriptional and epigenetic analysis in lineage-specific cell types. We anticipate that this versatile method can be used to study the mechanistic underpinnings of lineage specification, even when cell number is limiting and the presence of connective tissue is substantial.

## Materials and methods

### Commercial mouse strains

All animal experiments were approved by the Institutional Animal Care and Use Committee (IACUC) at UT Southwestern Medical Center and conformed to the National Institutes of Health (NIH) mandate for the care and use of laboratory animals. The *R26R*^*tdTomato/tdTomato*^ reporter (Strain number 007914), *Wt1*^*GFPCre*^ (stock number 010911), *Nkx2-5*^*IRES-Cre*^ (stock number 024637), *C57BL/6J* (in the study referred to as Wild type or WT) (stock number 000664), and B6;129-*Gt(ROSA)26Sor*^*tm5(CAG-Sun1/sfGFP)Nat*^/J (in the study referred to as Rosa26-Sun1-sfGFP-myc-Tag) (stock number 021039) mice were obtained from the Jackson Laboratory (Bar Harbor, ME).

### Antibodies

Anti-pericentriolar material 1 (PCM1) antibody (rabbit polyclonal, Atlas Antibodies, #HPA023370, dilution 1:200), anti-Myc antibody (rabbit polyclonal, Invitrogen, #PA1-981, dilution 2μg), and anti-phospholamban (PLN) antibody [2D12] (mouse monoclonal, Abcam, #ab2865, dilution 1:100) were used. The following fluorophore-conjugated secondary antibodies were used: Alexa Fluor 488 Goat Anti-Rabbit IgG (H+L) Antibody, 1:400 (Invitrogen), Alexa Fluor 555 Goat Anti-Mouse IgG (H+L) Antibody, 1:400 (Invitrogen), Alexa Fluor 647 Donkey Anti-Mouse IgG (H+L) Antibody, 1:400 (Invitrogen), and Alexa Fluor 647 Donkey Anti-Rabbit IgG (H+L) Antibody, 1:400 (Invitrogen).

### Nuclei isolation

Whole hearts were harvested and placed in cold 1X phosphate buffered saline (PBS) to remove excess blood. In a clean petri dish, tissue was added to freshly prepared ice-cold lysis buffer (0.32 M sucrose, 5 mM CaCl_2_, 3mM magnesium acetate, 2.0 mM EDTA, 0.5 mM EGTA, 10 mM Tris-HCl (pH 8.0), 1 mM DDT, 0.2% Triton-X-100) supplemented with protease inhibitor cocktail (Sigma). All subsequent steps were performed on ice. The tissue was minced thoroughly into tiny pieces. Next, the mixture was dounced with a type A pestle (Specification: 0.0035–0.0055 inches (0.089–0.14 mm), Kimble Chase, #885301–0002) exactly 18 strokes for adult P28 hearts and 5 strokes for P1 hearts in a glass douncer (2ml glass tissue grind tube, Kimble Chase, #885303–0002) followed by 2–3 strokes with type B pestle (Specification: 0.0010–0.0030 inches (0.025–0.076 mm), Kimble Chase, #885302–0002) to make a uniform suspension. The douncing step was optimized for heart tissue at all stages of postnatal development. The solution was put through two layers of cotton gauze and consecutively filtered with 100 and 70 μm nylon cell strainers (Falcon). The tubes were centrifuged for 8 minutes at 1000g at 4 °C, and the supernatant was carefully removed with a pipette from each tube and discarded.

Next, a density-gradient based ultracentrifugation was applied for separation of pure nuclei from other undesired cellular organelles. The crude nuclear pellets were resuspended in 4 ml of 2.1 M sucrose buffer (for CM nuclei) or 1.7 M sucrose buffer (for mixed nuclei) (Buffer composition for resuspension of crude nuclear pellet: 2.1 M/1.7 M sucrose, 3 mM magnesium acetate, 1 mM DTT, 10 mM Tris-HCl, pH 8.0, dissolved in nuclease free water). This was then layered onto 8 ml 2.2 M sucrose cushion (for CM nuclei) or 1.8 M sucrose cushion (for mixed nuclei) (Buffer composition for the bed/cushion onto which the resuspended crude pellet is loaded onto: 2.2 M/1.8 M sucrose, 3 mM magnesium acetate, 1 mM DTT, 10 mM Tris-HCl, pH 8.0, dissolved in nuclease free water), and centrifuged at 30,000g for one hour at 4°C. The supernatant was carefully discarded without disturbing the pure nuclei pellet to avoid contamination.

The pellet from each tube was then resuspended with 1 ml of nuclei resuspension buffer (NRB) (0.43 M sucrose, 70 mM KCl, 2 mM MgCl2, 10 mM Tris-HCl (pH 7.2), 5 mM EGTA). RNasin Plus RNase Inhibitor (Promega) at 80 U/ml was added to every buffer including the sucrose solutions for ultracentrifugation to minimize RNA degradation. Both spermine and spermidine (0.1M final concentration) were added to every buffer including the sucrose solutions for ultracentrifugation as DNA stabilizers. The NRB was supplemented with 10% glycerol to avoid clumping of nuclei. Precise yield of nuclei was measured using DAPI staining, followed by counting in chamber slide using Countess II FL (using DAPI light cube) Automated Cell Counter (Invitrogen). The 1.8 M sucrose cushion protocol that was optimized for heart nuclei isolation was implemented on the adult mouse kidney samples. The detailed buffer compositions are listed in S2–S4 Tables.

Before proceeding with any downstream applications, the quantity of the nuclear RNA that were extracted from input or pure nuclei (both 1.8M and 2.2M cushion purified nuclei) was measured by Qubit (Thermo Fisher Scientific) and genomic DNA quality and quantity was assessed by Agilent 4200 TapeStation system and Qubit (Thermo Fisher Scientific), respectively. These quality control steps indirectly provide information on the integrity of the nuclei content for use in downstream experiments and analyses.

### Immunostaining of nuclei

The pure nuclei pellet obtained at the end of ultracentrifugation was incubated with primary antibodies (1°Ab) at their respective dilutions in NRB + 10% Glycerol + DNA stabilizers + RNase Inhibitor for 30 minutes and very gently rocked on a Nutator. This was followed by centrifugation at 700g for 10 minutes. Next, the supernatant was carefully decanted, and the pellet was resuspended in NRB + 10% Glycerol + DNA stabilizers + RNase Inhibitor and incubated with appropriate fluorophore-conjugated secondary antibodies (2°Ab) for 45 minutes in the dark with gentle agitation on a Nutator. The nuclei suspension was washed for 10 minutes by centrifuging at 700g. Without disturbing the pellet, the supernatant was cautiously discarded. For confocal imaging of the immunolabeled nuclei, a small volume from the nuclei + 1°Ab + 2°Ab complex was mounted with Vectashield containing DAPI (Vector Labs). All steps were performed in a cold room.

### Cell line for bead validation for magnet-assisted nuclear immunoprecipitation (MAN-IP)

COS7 (Catalog #ATCC CRL-1651, African green monkey kidney fibroblast-like cell line) was used for performing experiments to validate and use the optimally performing beads. Protein G Dynabeads (ThermoFisher Scientific, #10003D) did not perform with high specificity and sensitivity as previously reported^17^ when used with tagged cardiac nuclei. Therefore, Anti-Rabbit IgG Microbeads (Miltenyi) were used for all experiments due to its superior performance.

### MAN-IP

Following 30 minute incubation with primary antibody and centrifugation at 700g spin for 10 minutes, the nuclei + 1°Ab complex in the pellet was resuspended in 80μl of MACS Buffer composed of 1X PBS (Tissue Culture grade; Ca^2+^, Mg^2+^ free), 0.5% Nuclease free Bovine Serum Albumin (BSA), and 2mM EDTA. Next, 20 μl of Anti-Rabbit IgG Microbeads (Miltenyi) was added with fluorophore-conjugated 2°Ab (dilution 1:400), mixed, and incubated for 20 minutes in the refrigerator in the dark. This was followed by a wash with 1ml of MACS buffer at 300g for 10 minutes at 4°C. Subsequently, the recommended protocol for Anti-Rabbit IgG Microbeads-mediated magnetic separation/enrichment of immunolabeled nuclei was performed with MACS MS columns (Miltenyi). Both the flow through (FT) and eluate fractions were collected and mounted with Vectashield + DAPI (Vector Labs) on glass slides to visualize with a confocal microscope for evaluating sensitivity, specificity, and fold enrichment of the MAN-IP assay.

### Confocal microscopy

Confocal images of immunostained nuclei were acquired with a Nikon A1R+ scanning confocal system. Objectives used were 10X and 20X (dry). Excitation laser filters used were 488 nm line, green fluorescence; 555 nm line, red fluorescence; and 647 nm line, far-red fluorescence. Images were analyzed using NIS Elements Viewer v4.2 software, ImageJ, and Adobe Photoshop CS6 Extended software.

### RNA extraction and real-time quantitative Polymerase Chain Reaction (qPCR)

The nuclei suspension (input, eluate/enriched fraction, etc.) was mixed with equal volume of DNA/RNA Shield (2X concentrate provided in ZR-Duet DNA/RNA MiniPrep Plus kit, #D7003), and RNA was extracted using the same kit following the instruction manual. Qubit quantification was used to estimate RNA concentration. Next, RNA was concentrated to 10μl volume using the RNA Clean and Concentrator-5 Kit (Zymo Research, #R1015). The RNA was converted to cDNA using SuperScript III Reverse Transcriptase (Invitrogen) with random priming. The synthesized cDNA was diluted 1:10 and used as template for the subsequent qPCR. Real time qRT-PCR assay was carried out in ABI PRISM 7000 Sequence Detection System (Applied Biosystems) using Azura Quant Green Fast qPCR Mix HiRox recommended protocol. qPCR primer sequences are provided in S1 Table.

### Statistical analysis

Statistical calculations were performed using GraphPad Prism 7 software. Details related to statistical testing are described in each figure legend. Graphs were also made using GraphPad Prism 7 software.

### ATAC-Seq library preparation and sequencing

Two independent biological replicates per experimental sample were generated for library construction. To generate ATAC-Seq libraries, the improved Omni-ATAC-Seq protocol, which is detailed in study referred to in [27] was used. Briefly, 50,000 nuclei were pelleted in ATAC-Resuspension Buffer (recipe published in the same study) containing 0.1% Tween-20 after counting nuclei with a Countess II FL (using DAPI light cube) Automated Cell Counter. The supernatant was carefully discarded, and the nuclei pellet was incubated with transposase. Transposed DNA was cleaned with Zymo DNA Clean and Concentrator-5 Kit (#D4014). This was followed by pre-amplification of transposed fragments, qPCR amplification, and KAPA library quantification to determine additional cycles for the final PCR amplification (none to very few additional cycles were required with the Omni-ATAC-Seq protocol). After final amplification, the ATAC-Seq library was cleaned with Zymo DNA Clean and Concentrator-5 Kit (#D4014) and submitted for Tape Station and Qubit analysis. 4nM of each ATAC-Seq library was used for paired-end sequencing on an Illumina NextSeq 500 Mid Output (130 M) flow cell. Image analysis and base calling were performed with the Sanger/Illumina 1.9 pipeline.

### Bioinformatic analysis for ATAC-Seq datasets

Raw FASTQ files were analyzed using FastQC v0.11.5 [28] and FastQ Screen v0.11.4 [29], and reads were quality-trimmed using fastq-mcf (ea-utils/1.1.2–806) [30]. The trimmed reads were mapped to the mm10 assembly of the mouse genome (the University of California, Santa Cruz, version from igenomes) with Bowtie2 (version 2.3.3.1) [31]. The duplicates were marked using picard-tools (v2.2.1) and blacklist regions were removed using bedtools (v2.7.1) [32]. Tn5 shifting of bam files was performed using the open-source Perl script "ATAC_BAM_shiftrt_gappedAlign.pl" [33]. The ATAC-Seq peaks were called using MACS2 (version 2.1.0.20160309) [34] with a q-value threshold of 0.05 and using random background. Furthermore, we divided the fragments into nucleosome free (sub-nucleosomal, <100 bp) and nucleosome occupied or bound fragments (>100 bp). After peak calling, we merged overlapping peaks from replicates (bedtools [32] merge) to yield a peak set for each sample. We used bedtools intersect to get common regions between peak files of replicates of the same experimental group. The annotation of peaks was done using ChIPseeker [35]. The genome-wide distribution of ATAC-Seq regions on promoters, exons, introns, and intergenic regions in each of the samples was also done using ChIPseeker [35]. For ATAC-Seq in model organisms, the peak file (NAME_peaks.narrowPeak) can be uploaded directly to the UCSC genome browser for generating the browser tracks and ENCyclopedia of DNA Elements (ENCODE) peaks were added by configuring the UCSC Genome Browser Track to import public track hubs, in this case ENCODE DHS-Seq dataset [36] from P1 mouse heart (University of Washington, (Replicate 1 file name: GSM2195800_ENCFF539AJY_signal_of_unique_reads_mm10.bigWig and Replicate 2 file name: GSM2195801_ENCFF032ZEL_signal_of_unique_reads_mm10.bigWig)). Web-based PANTHER Gene Ontology (GO) tool was used for generating GO terms for nearest neighbor genes for peaks that were common to two replicates of each ATAC-Seq sample. We generated Pearson correlation graphs as well as Principle Component Analysis (PCA) plots, heatmaps, and profile plots for the ATAC-Seq and DHS-Seq samples using deepTools2 [37].

## Results

### Overview of PAN-INTACT protocol development

**Fig 1:**
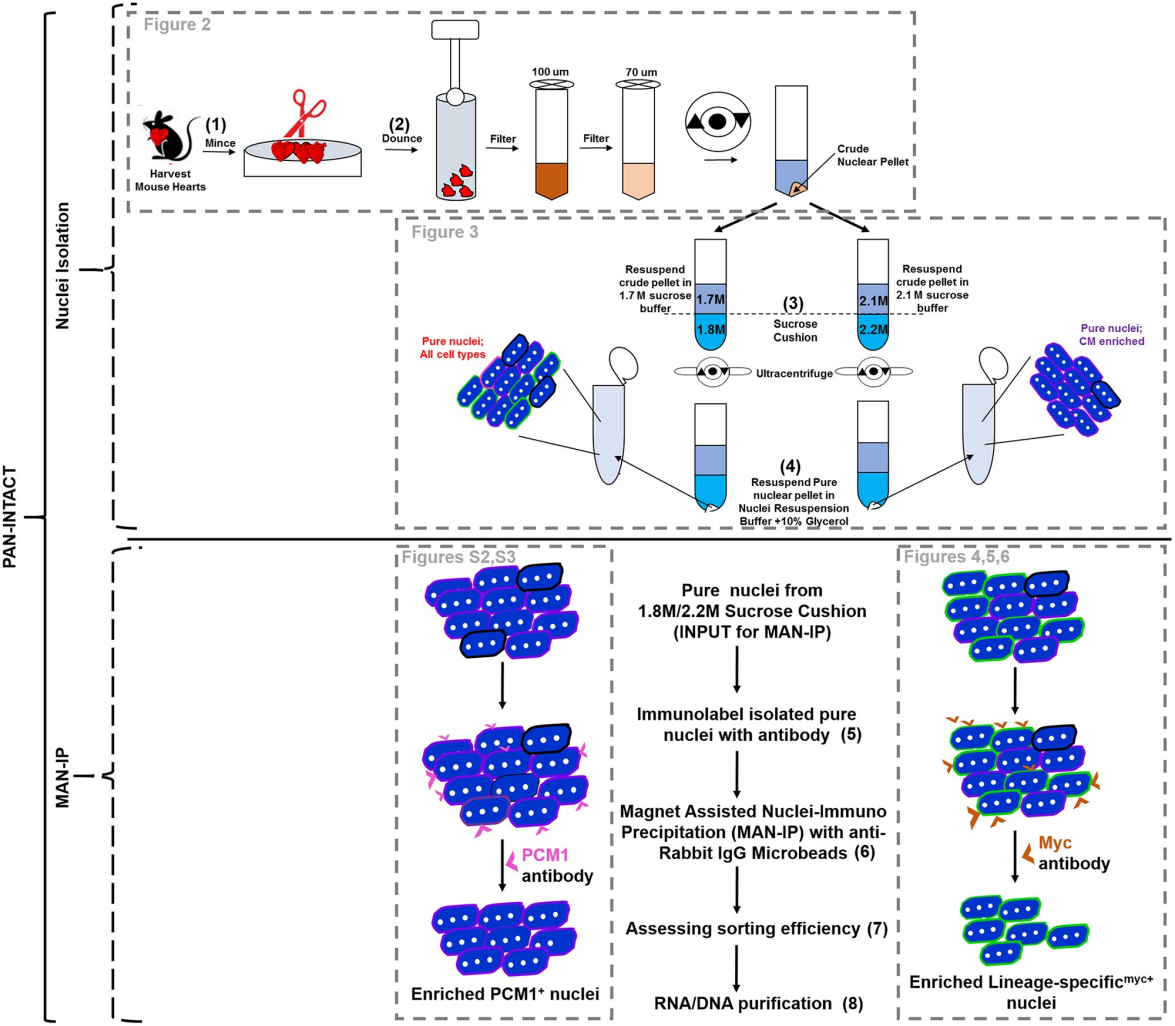
PAN-INTACT overview. Schematic showing the overall PAN-INTACT workflow. Mouse hearts (or other tissues) are harvested and minced extensively before douncing. Homogenized tissue is passed over sequential filters, and a crude nuclear pellet is obtained following centrifugation. The crude pellet is layered on a sucrose cushion, and ultracentrifugation yields a pure nuclear pellet, which is resuspended in buffer supplemented with 10% glycerol to avoid clumping. Pure nuclei are incubated with an appropriate antibody and purified further by magnet-assisted nuclei immunoprecipitation (MAN-IP) using anti-rabbit IgG Microbeads. The protocol is broadly split into nuclei isolation and MAN-IP segments, and the dashed boxes designate optimization steps that are shown in the indicated figures.

PAN-INTACT is a versatile method for isolating cell type specific nuclei from complex, fibrous tissues, such as the adult heart. A key advantage of our method is the wide applicability to study both abundant and rare cell types at the transcriptomic and epigenomic level with high resolution. Following organ removal (Fig 1), the tissue is extensively minced with scissors and dounced with a pestle. Sequential filtering of ruptured cellular contents is followed by centrifugation to pellet released nuclei. The crude nuclear pellet is then resuspended in sucrose and layered on a sucrose cushion. Following ultracentrifugation, the pure nuclear pellet is resuspended in a stabilizing buffer supplemented with 10% glycerol to prevent clumping of nuclei. The resuspended nuclei are then incubated with the appropriate antibody and subjected to magnet assisted nuclei immunoprecipitation (MAN-IP) to isolate cell type specific nuclei for downstream analysis. To develop PAN-INTACT, we sought to identify the most efficient, cost-effective, and versatile method for cell type specific isolation of nuclei from intact mouse organs. Individual components of the overall PAN-INTACT protocol are boxed and discussed in subsequent sections (Fig 1).

### Optimization of cardiomyocyte (CM) nuclei extraction

**Fig 2:**
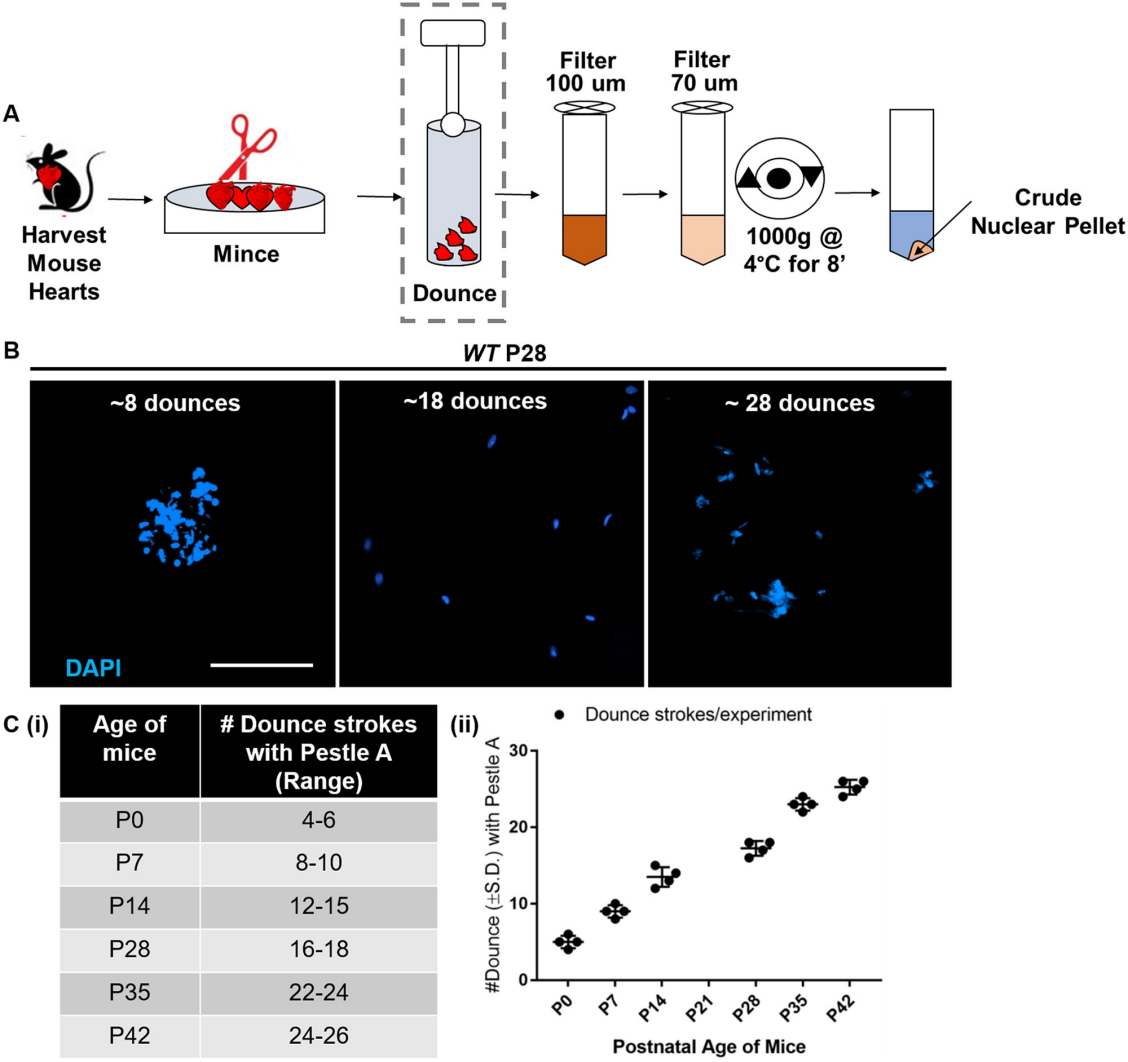
Optimization of nuclei extraction from mouse hearts by dounce homogenization. A) Diagram detailing the PAN-INTACT steps involved in nuclei extraction from heart tissue with the dashed box highlighting the douncing step that underwent optimization. B) Hearts derived from mice of varying postnatal age were systematically assessed for the ideal number of douncing strokes to release nuclei (stained with DAPI). For P28 hearts, 18 dounces were optimal. Under-douncing (8 strokes) led to incomplete nuclei release, while over-douncing (28 strokes) led to nuclei fragmentation. Scale bar: 200 μm. C) The optimal range of douncing strokes required to release nuclei at various postnatal ages is shown as a table (i) and graph (ii) with Standard Deviation (S.D.).

Isolation of intact nuclei from mouse tissue requires organ dissection, tissue homogenization, and release of nuclei (Fig 2A). Although dissection tecniques are well-established for each organ system, many alternative methods for tissue homogenization and nuclei release exist. Thus, we sought to optimize these methods for fibrous adult heart tissue as a proof-of-principle. For fixed heart tissue, efficient homogenization can be accomplished using a blender and hand-held homogenizer. Although effective, this strategy necessitates the purchase of expensive equipment. Mo and collegues utilized inexpensive razor blade mincing for homogenization [22], but brain tissue is much softer than adult heart tissue. In preliminary studies, we evaluted the use of a hand-held homogenizer for adult heart tissue, but excessive frothing of the solution occurred if the tissue was not extensively minced beforehand. Based on this observation, we instead minced the heart tissue extensively with fine-tipped scissors and observed uniform tissue homogenization without the use of a motorized device. Thus, we concluded from these preliminary studies that extensive mincing was sufficient to homogenize fibrous adult heart tissue without costly additional equipment.

Douncing with a pestle is a well-established method for releasing nuclei from a cell suspension in hypotonic solution [38]. This approach obtains a high percentage of cell nuclei and mitochondria from tissues, while minimizing heat produced by friction since the pestle is submerged in buffer. Finely minced tissue pieces undergo initial grinding using the "loose" (Type A) pestle. Pestle A is a large clearance pestle that is required for initial sample reduction and disruption of connective tissue. Then, a “tight” (Type B) pestle is used to ensure complete disruption of extracellular connective tissue. Fundamentally, Pestle B is the small clearance pestle that forms the final homogenate. For more fibrous tissues like the heart, douncing with Pestle A (large clearance) is crucial to reduce the fibrous tissue pieces into a smooth suspension. The last 2–3 dounces with Pestle B release the crude nuclei. To apply this technique to fresh tissue, douncing parameters must be individually optimized for each homogenized tissue. This is a key challenge for fibrous tissues and particularly for those whose mechanical properties change with development and/or maturation, such as the heart. In contrast, the stiffness of the brain does not change significantly after birth. To isolate intact nuclei from mouse hearts across all stages of development, therefore, we carefully optimized and modified existing protocols.

We initially focused on the number of strokes with the type A pestle, noting the number of douncing strokes used in previous studies for extracting nuclei from heart (8) and brain (5) as a starting point. We dissected and homogenized hearts from P28 mice and subjected them to variable douncing strokes with a type A pestle. For each sample, the quality of the nuclei was assessed by DAPI staining and confocal microscopy to carefully examine nuclear morphology. Whereas under-douncing (8 strokes) resulted in poor yield due to aggregation of nuclei, over-douncing (28 strokes) led to low yield due to nuclei rupture (Fig 2B). In contrast, optimal douncing (18 strokes) led to well-dispersed nuclei with preserved morphology (Fig 2B). To quantitatively evaluate the relationship between douncing and ages of mouse hearts, the number of morphologically intact nuclei was estimated using a Countess II FL (using DAPI light cube) Automated Cell Counter. We assigned optimal douncing strokes at each sample age (Fig 2Ci and 2Cii) when we observed more than 90% of the nuclei were well-dispersed and intact by DAPI visualization using confocal microscopy (Fig 2B). Using this approach, we experimentally established the range of the number of douncing strokes required for efficient nuclei release at postnatal ages P0 to P42 (Fig 2C). For the type B pestle, we found that 2–3 strokes worked best, as described by Bergmann and colleagues, regardless of age.

Collectively, these data establish optimal parameters for extraction of nuclei from cardiac tissue of varying age. Interestingly, we observe a direct relationship between the number of douncing steps required for efficient release of nuclei and age, which is consisent with the known age-dependent increase in heart stiffness [39]. Based on our experience optimizing nuclei release from cardiac tissue, we noted that the resistance encountered by the pestle dramatically decreased at the point of maximal nuclei extraction. In turn, this knowledge was applied to quickly determine the ideal parameters for extracting nuclei from kidney, another fibrous organ (see below). Importantly, proper extraction of nuclei is a crucial step to maintain RNA and DNA integrity for downstream analysis.

### Evaluation of magnetic bead source for CM nuclei immunoaffinity purification

Nuclei can be isolated by flow cytometry or bead-based immunoaffinity purification [7, 9, 10, 17, 23–26]. For our protocol development, we decided to pursue bead-based purification to avoid the need for a dedicated flow cytometry facility. Purification of nuclei from CMs and neurons has been performed with anti-IgG Microbeads and Protein G Dynabeads, respectively [10, 17, 25, 26]. Therefore, to determine whether Protein G Dynabeads could provide a favorable alternative for purification of CM nuclei, we initially performed nuclei mixing experiments to test the specificity of capture. Given that PCM1 can label CMs, we asked whether Protein G Dynabeads could successfully purify CMs away from a contaminating cell type, a task that resembles our overall goal. Therefore, we mixed CM nuclei with COS7 nuclei, which are easy to identify based on their larger size (S1Ai and S1Aii Fig), at a ratio of 1:100 (S1Aiv Fig) and performed PCM1-based immunoaffinity purification. As a control, we applied the same purification scheme to CM nuclei alone. Although Protein G Dynabeads were capable of attaching to PCM1-labeled CM nuclei (S1Aiii Fig), they also non-specifically interacted with COS7 nuclei in the mixed population (S1Aiv Fig). Based on this observation, we decided to develop our protocol with anti-IgG Microbeads, which have been used to successfully isolate CM nuclei [10, 25, 26].

PCM1 labels CM nuclei from mouse and human hearts, and this unique labeling characteristic has allowed investigators to profile epigenetic marks and nuclear RNA in a cardiomyocyte-specific manner [10, 25, 26]. To test whether the anti-IgG Microbeads were compatible with our nuclei extraction method, we performed immunoaffinity purification by overnight incubation with PCM1 antibody as described previously^9^ (S1B Fig). When applied to nuclei from P28 mouse hearts, this protocol successfully isolated PCM1^+^ nuclei and left behind PCM1^-^ nuclei in the flow through (FT) (S1C Fig). We used two parameters to evaluate the efficiency of the MAN-IP method throughout our study. Specificity is defined here as the percentage of Label^+^ nuclei over total nuclei in the eluate. In contrast, sensitivity represents the percentage of Label^+^ nuclei in the eluate over the total Label^+^ nuclei in the original sample. In other words, sensitivity describes how well the assay captures all Label^+^ nuclei, while specificity describes how accurate the assay performs to only capture Label^+^ nuclei. Quantification of these observations determined that our nuclei extraction method in combination with antibody-based purification of PCM1^+^ nuclei achieves a specificity of ≥ 97% (S.D. = ±0.707) (nearly all nuclei in eluate were GFP^+^) and a sensitivity of ≥95% (S.D. = ±0.5) (very few GFP^+^ nuclei were detected in the FT) (S1D Fig). These observations were confirmed in four independent experiments (S1D Fig with Range in percentage ± S.D.) by analyzing at least 100 nuclei per experiment, and they compare favorably with previous studies [9, 10, 25, 26].

### Modulating the sucrose cushion enables rapid, label-free nuclei purification and preserves nuclei heterogeneity

**Fig 3:**
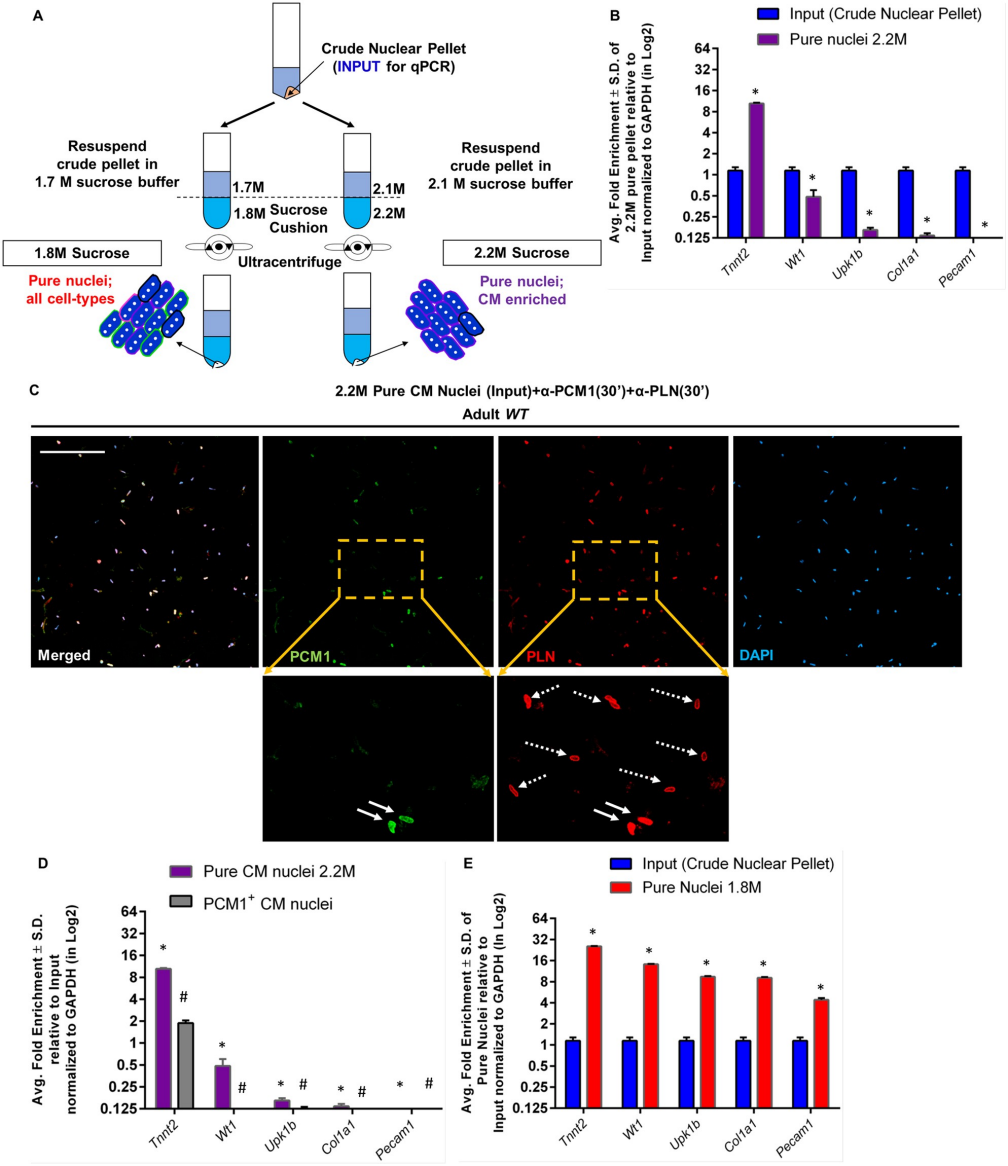
Altering the density of the sucrose cushion enriches for CM or heterogenous cardiac nuclei. A) Diagram detailing the PAN-INTACT steps involved in nuclei purification using two alternative sucrose cushions. B) Following nuclei purification over a 2.2M sucrose cushion, qRT-PCR was performed. Enrichment of CM nuclei was demonstrated by increased Troponin T (Tnnt2) expression in purified versus input (crude nuclear pellet). In contrast, epicardial (Wt1/Upk1b), fibroblast (Col1a1), and endothelial (Pecam1) markers were not enriched. * indicates p-value < 0.05 compared with input (crude nuclear pellet). C) Nuclei purified over a 2.2M sucrose cushion were immunolabeled with PCM1 (green) and Phospholamban (PLN; red) antibodies with DAPI counterstaining (blue). Nearly every DAPI^+^ nucleus is PLN^+^, while PCM1 labels only a subset of purified nuclei. Zoomed images are provided for detailed visualization (solid arrow, PCM1^+^/PLN^+^ nuclei; dashed arrow, PCM1^-^/PLN^+^ nuclei). Scale bar: 100μm. D) Cardiac nuclei were isolated over a 2.2M sucrose cushion. Half of the purified nuclei were then subjected to PCM1-based immunoaffinity purification for 30 minutes. PCM1^+^ nuclei were directly compared to input nuclei to determine cardiac enrichment by performing qRT-PCR with lineage-specific markers: Tnnt2 (CM), Wt1/Upk1b (epicardial cell), Col1a1 (fibroblast), and Pecam1 (endothelial cell). * and # indicate p-value < 0.05 for Pure CM nuclei 2.2M and PCM1^+^ CM nuclei, respectively, compared with input. E) Following nuclei purification over a 1.8M sucrose cushion, qRT-PCR was performed. Enrichment of cardiomyocyte, epicardial, fibroblast, and endothelial markers was observed. * indicates p-value < 0.05 compared with input (crude nuclear pellet). (B, D, and E) Y-axis scale: Log2; Gapdh was used as a reference gene to normalize qRT-PCR data, which is represented as average fold enrichment ± S.D. for reactions done in triplicate.

Purification of nuclei by gradient centrifugation eliminates unwanted cellular debris from the preparation. To apply INTACT to neuronal subtypes, Mo and colleagues purified nuclei over an iodixanol gradient [17], but iodixanol is relatively expensive. Alternatively, Bergmann and colleagues purified nuclei over a 2.2M sucrose cushion prior to immunoaffinity isolation of CM nuclei with a PCM1 antibody [9, 23, 24]. For our PAN-INTACT protocol, we used a 2.2M sucrose gradient as a starting point to optimize nuclei purification from adult mouse hearts (Fig 3A).

The adult heart is composed of multiple cell types, including CMs, endothelial cells, epicardial cells, endocardial cells, fibroblasts, and other cell types. Therefore, we presumed that nuclei purification over a 2.2M sucrose gradient should yield approximately ~30–40% CM nuclei based on current estimates of mammalian heart cellular composition [40, 41]. To directly test this assumption, we purified nuclei from P28 mouse hearts over a 2.2M sucrose cushion by ultracentrugation. RNA was then isolated from the purified nuclei and converted into cDNA for qRT-PCR anlaysis. Surprisingly, we found that RNA isolated from these nuclei was enriched for genes expressed in cardiomyocytes (Tnnt2) but not epicardial cells (Wt1/Upk1b), fibroblasts (Col1a1), or endothelial cells (Pecam1) (Fig 3B). The input used for calculating enrichment of gene expression in Fig 3B was crude nuclear pellet that was not yet purified over sucrose cushion. To confirm these observations, nuclei were labeled for 30 minutes with PCM1 and Phospholamban [26] (PLN) antibodies and counter-stained with DAPI. The stained nuclei were placed on a coverslip and visualized by confocal microscopy (Fig 3C). We observed that only a fraction of the nuclei stained for PCM1, while, remarkably, nearly all the nuclei stained for PLN. Taken together, these results demonstrate that 2.2M sucrose gradient purification results in a nearly uniform population of CM nuclei based on PLN staining. Interestingly, our findings also show incomplete CM nuclei staining by PCM1, suggesting that PCM1 does not efficiently label all CM nuclei under our assay conditions.

Although PCM1 has been widely used to stain and isolate CM nuclei [9, 10, 24–26], certain disadvantages of this approach deserve mention. First, PCM1 antibody staining of nuclei is typically performed overnight, which necessitates prolonged handling times for immunoaffinity purification. Second, although PCM1 is distributed uniformly around the nucleus in post-mitotic cells [26], it is particularly enriched at centrioles in proliferating cells [42]. Third, we repeatedly observed that short incubation times with PCM1 antibody resulted in sub-optimal labeling, which suggests that the PCM1 antibody has a particularly low avidity for its cognate epitope (Fig 3C). Based on these considerations, a method for isolating CM nuclei that requires shorter handling times and ensures isolation of both dividing and non-dividing CMs would be particularly attractive.

Since our results suggested that 2.2M sucrose gradient purification yielded a nearly pure population of CM nuclei (Fig 3B), we wondered whether this step alone was sufficient to isolate CM nuclei for downstream analysis. Therefore, we performed a side-by-side comparison between nuclei isolated over a 2.2M sucrose cushion alone or followed by rapid (30 minutes) PCM1 antibody-based immunoaffinity purification. To estimate the efficiency of CM nuclei isolation, we performed qRT-PCR for marker genes of specific cell types. From this experiment, we found that CM transcripts were highly enriched in nuclei isolated over the 2.2M sucrose cushion and compared favorably with nuclei isolated by PCM1-based immunoaffinity purification (Fig 3D). Additionally, we note a minor contamination of the Wt1 epicardial marker in the 2.2M sucrose gradient sample with a nearly complete absence of epicardial, fibroblast, and endothelial markers (Fig 3D). Taken together, we conclude that, for most applications, nuclei purification over a 2.2M sucrose cushion is sufficient to yield a nearly homogenous population of CM nuclei. In particular, this CM nuclei purification method has the added advantage of minimal handling time, thereby improving nuclear RNA and DNA quality for downstream analyses. Finally, since neither antibody purification nor a genetic tag are required, this strategy for nuclei isolation is ideally suited for human heart tissue.

Since our overall study objective was to develop a versatile method for isolation of cell type specific nuclei, we next sought to modify the sucrose gradient step in order to preserve as much nuclear diversity as possible. Based on a previous report demonstrating that a 1.8M sucrose cushion results in better yields and morphological preservation of nuclei [43], we decided to apply this modification to the isolation of nuclei from heart tissue (Fig 3A). Since nuclear markers for non-CMs are not known, we used qRT-PCR for specific marker genes to assess the cell type heterogeneity of the purified nuclei. We isolated RNA from the purified nuclei and performed qRT-PCR using primers for specific marker genes. We found that nuclei purified over a 1.8M sucrose cushion yielded RNA enriched for marker genes of CMs (Tnnt2), epicardial cells (Wt1/Upk1b), fibroblasts (Col1a1), and endothelial cells (Pecam1) (Fig 3E). This is in stark contrast to our results with the 2.2M sucrose cushion (Fig 3B). The input used for calculating enrichment of gene expression in Fig 3E was the crude nuclear pellet prior to purification over sucrose cushion (Fig 3A). To directly compare the cell type distribution of nuclei obtained from isolation over 1.8M and 2.2M sucrose cushions, we performed qRT-PCR for specific marker genes (S2 Fig). In these experiments, we used either whole heart cDNA (S2A Fig) or crude nuclear pellet (S2B Fig) as the reference group. Although the absolute levels of enrichment differ slightly, these data demonstrate representation of heterogeneous nuclei in the 1.8M sample and relatively homogeneous CM nuclei in the 2.2M sample. Collectively, these data demonstrate successful preservation of nuclei heterogeneity following centrifugation over a 1.8M sucrose cushion as opposed to nearly uniform isolation of CM nuclei after 2.2M sucrose gradient purification.

To rule out any effect of sucrose-gradient modulation on nuclei yield, the quantity of pure nuclei obtained after 2.2M sucrose-gradient and 1.8M sucrose-gradient purification was tabulated from multiple experiments across different stages of mouse development (S5 Table). We observed a trend towards of higher yields of nuclei for 1.8M sucrose gradient purification (heterogenous nuclei from major cardiac cell-types) relative to a 2.2M sucrose cushion (primarily CM nuclei only).

### PAN-INTACT successfully isolates Nkx2-5 lineage positive cardiac nuclei

**Fig 4:**
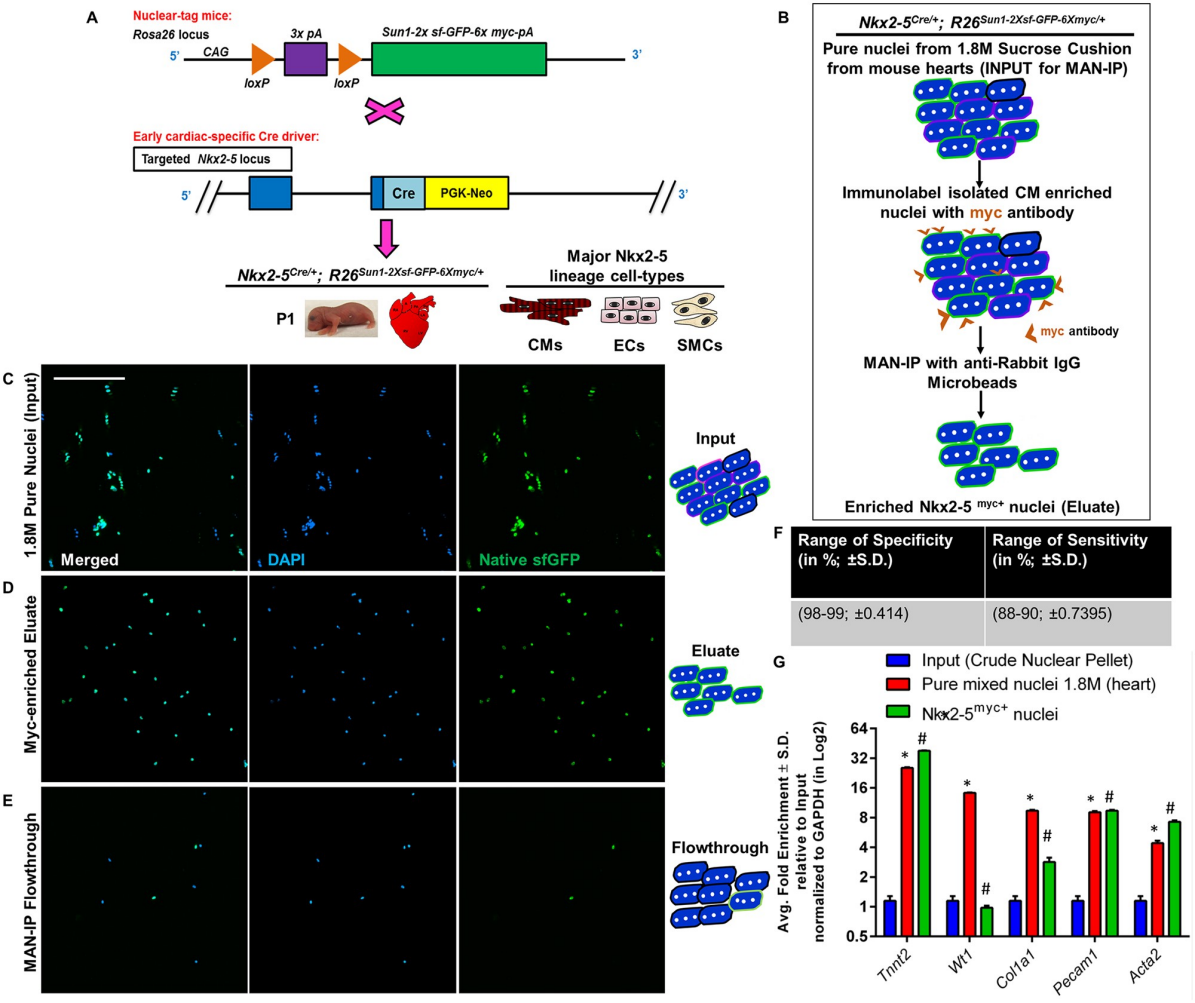
Efficient isolation of Nkx2-5 lineage positive cardiac nuclei using PAN-INTACT. A) Schematic of the breeding strategy used for these experiments. Crossing of R26^Sun1-2xsf-GFP-6xmyc/+^ and Nkx2-5^Cre/+^ mice led to Nkx2-5^Cre/+^; R26^Sun1-2xsf-GFP-6xmyc/+^ offspring that drive expression of the SUN1 fusion protein in the nuclear membrane of Nkx2-5 lineage positive cells. The major Nkx2-5 positive lineages in the heart include CMs, smooth muscle cells (SMCs), and endocardial cells (ECs). B) Experimental workflow for magnet-assisted nuclear immunoprecipitation (MAN-IP). C-E) Heterogeneous cardiac nuclei were purified from P1 mouse hearts using a 1.8M sucrose cushion followed by Myc MAN-IP. Confocal images of DAPI-stained nuclei showing native sfGFP expression in input nuclei (C), MAN-IP eluate (D), and MAN-IP flow-through (E). Magnification 100μm. F) Quantification of labeled nuclei from four independent experiments (n = 100 nuclei per experiment) for Myc MAN-IP of Nkx2-5 lineage positive cardiac nuclei was used to calculate specificity and sensitivity (range in percentage with S.D. in parentheses). G) qRT-PCR was performed on purified Nkx2-5 lineage positive nuclei and pure mixed nuclei compared with input. Gapdh served as an internal standard for qPCR, and the data are represented as average fold enrichment ± S.D. of triplicate reactions for each marker gene over input. * and # indicate p-value < 0.05 compared with input. Y-axis scale: Log2.

Having established a protocol for extracting cardiac nuclei, we next sought to apply the INTACT method to a specifically labeled lineage in the mouse heart. Mouse INTACT utilizes the R26^Sun1-2xsf-GFP-6xmyc/+^ line, which harbors a floxed-stop cassette followed by a Myc-tagged GFP-fusion protein that localizes to the nuclear membrane [17]. Following Cre recombination, the fusion protein is expressed, and the nuclei of Cre^+^ cells can be isolated by immunoaffinity purification using a Myc or GFP antibody. To demonstrate application of INTACT to heart tissue, we bred R26^Sun1-2xsf-GFP-6xmyc/+^ mice with the Nkx2-5^Cre/+^ driver line [44] (Fig 4A). The Nkx2-5^Cre/+^ line labels multipotent cardiac progenitor cells that give rise to CMs, smooth muscle cells, and endothelial cells [45,46]. Following Cre-mediated recombination, expression of the SUN1-sfGFP-myc fusion protein in Nkx2-5 lineage positive nuclei facilitates their isolation using Myc antibody precipitation (Fig 4B).

We initially chose to utilize Nkx2-5^Cre/+^ mice to mark the first heart field (Nkx2-5 lineage-positive) lineage within the heart. In this way, we could profile descendants of Nkx2-5 lineage-positive progenitors. Given that Nkx2-5 lineage-positive cells consist of multiple cell types (CMs, smooth muscle cells, and endothelial cells) [45,46], we used the 1.8M sucrose cushion protocol to purify mixed cardiac nuclei from Nkx2-5^Cre/+^; R26^Sun1-2xsf-GFP-6xmyc/+^ mice at P1. Then, immunoaffinity purification with a Myc antibody could be used to separate Nkx2-5 lineage-positive CM, smooth muscle cells, and endothelial cell nuclei away from the nuclei of other non-CMs within the heart, such as fibroblasts and immune cells. Nuclei were stained with DAPI for visualization by fluorescence confocal microscopy (Fig 4C–4E). We observed that nearly all nuclei in the eluate were GFP^+^ (Fig 4D), thus demonstrating successful immunoaffinity purification. In contrast, the FT contained very few GFP^+^ nuclei (Fig 4E) compared with either the eluate (Fig 4D) or the input fraction (Fig 4C). Based on quantification of confocal images from four independent experiments (n = 100 nuclei per experiment), we calculated a specificity of ≥98% (with S.D. = ±0.414) and a sensitivity ≥88% (with S.D. = ±0.7395) for Myc-antibody MAN-IP for isolation of Nkx2-5 lineage positive cardiac nuclei (Fig 4F).

To directly assess the cell type identity of purified nuclei, we performed qRT-PCR analysis for specific marker genes. We confirmed that Nkx2-5 lineage positive nuclei express markers of CMs (Tnnt2), smooth muscle cells (Acta2), and endothelial cells (Pecam1) with a minor contamination from fibroblasts (Col1a1) (Fig 4G). In addition, we stained and imaged input nuclei with Myc, PCM1, and PLN antibodies (S3A–S3C Table). As expected, all GFP^+^ nuclei were also Myc^+^ (S3A Fig). However, only a subset of GFP^+^ nuclei were PCM1^+^ (S3B Fig) or PLN^+^ (S3C Fig), which is consistent with the fact that PCM1 and PLN only stain CMs, whereas Nkx2-5 marks additional cells types. To confirm this conclusion, we performed the same staining experiments on input nuclei derived from a 2.2M sucrose cushion (S3D–S3F Fig), which consists of nearly pure CM nuclei. We found that Myc (S3D Fig) and PLN (S3F Fig) antibodies stained all nuclei, thus confirming that the Nkx2-5 lineage efficiently marks CM nuclei. In contrast, PCM1 stained only a fraction of the total CM nuclei (S3E Fig), which is consistent with our prior observations (Fig 3C). Altogether, these data demonstrate successful application of INTACT to heart tissue for the isolation of Nkx2-5 lineage positive cardiac nuclei.

### Nuclei obtained by PAN-INTACT generate high-quality chromatin accessibility maps

**Fig 5:**
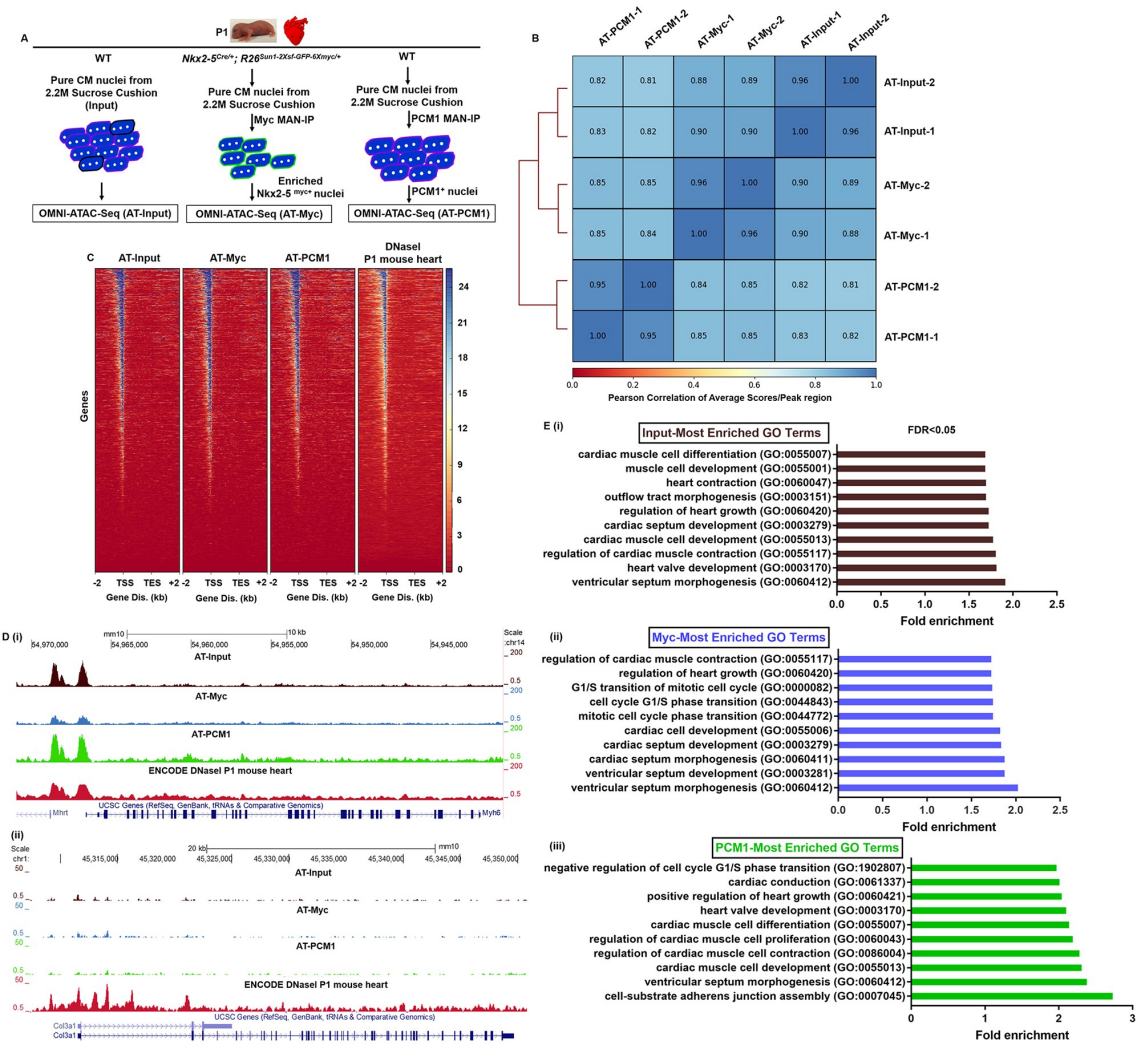
PAN-INTACT enables generation of high-resolution chromatin accessibility maps. A) Schematic diagram of experimental setup. CM nuclei were isolated from WT and Nkx2-5^Cre/+^; R26^Sun1-2xsf-GFP-6xmyc/+^ P1 mouse hearts using 2.2M sucrose-cushion, an aliquot of which was used as the input sample. Remaining nuclei were subjected to MAN-IP using a Myc or PCM1 antibody. Chromatin accessibility maps were generated for each sample individually using the OMNI-ATAC-Seq protocol. B) Similarity matrix demonstrating high pairwise Pearson correlations for ATAC-Seq biological replicates and amongst different samples. C) Heatmap showing a high degree of overlap for accessible sites within input, PCM1, and Myc samples. As an additional comparison, DHS-seq data is shown for P1 mouse heart from the ENCODE dataset. Kb (Kilobases) D) Genome browser tracks for canonical markers of CMs (i, Myh6) and fibroblasts (ii, Col3a1) with ATAC-seq peaks for each sample and DHS-seq peaks for comparison. E) Gene Ontology (GO) analyses for annotated nearest neighboring genes surrounding ATAC-seq peaks from all 3 samples (i-iii) display terms related to cardiac growth and function. Displayed results represent GO terms only with False Discovery Rate (FDR) < 0.05.

Given that PAN-INTACT successfully isolates cell type specific nuclei from cardiac tissue at different developmental ages for gene expression analysis, we next focused on coupling nuclei isolation with assays of genomic architecture. Thus, we evaluated whether OMNI-ATAC-Seq could be applied to purified Nkx2-5^+^, PCM1^+^, and 2.2M sucrose cushion isolated input nuclei from postnatal day 1 mouse hearts (Fig 5A). We chose to use the 2.2M sucrose cushion in these studies for two reasons. First, since we showed that the 2.2M sucrose cushion successfully isolates CM nuclei, this would allow us to directly compare ATAC-Seq maps generated by PCM1 MAN-IP and sucrose cushion purification alone. Second, we did not want to contaminate the ATAC-Seq maps for Nkx2-5^+^ nuclei with non-CM accessibility peaks, since it is well-established that the Nkx2.5 lineage also gives rise to cardiac smooth muscle and endothelial cells. Inclusion of these additional cells would minimize our ability to directly compare the three independent samples.

ATAC-Seq was performed under each condition in duplicate, and next generation sequencing (NGS) of the resulting libraries yielded a range of 12–55 million mapped reads per library (S6 Table). Using the collective sequencing data, we performed unsupervised hierarchical clustering to derive a Pearson correlation matrix (Fig 5B). In addition to replication across biological samples, we observed a strong correlation between different samples. Principle component analysis (PCA) also demonstrated a strong correlation amongst replicates (S4A Fig). Interestingly, the PCA results suggest that the Nkx2-5^+^ and PCM1^+^ datasets cluster more closely together than the input dataset, hinting at a common biological property of Nkx2-5^+^ and PCM^+^ nuclei that are distinct from the overall CM population. Nevertheless, these data collectively demonstrate high correlation amongst and between all samples analyzed, which is consistent with their origin from a common cell type (CMs).

As demonstrated previously, high-quality ATAC-Seq datasets generate accessibility profiles with a reproducible size distribution and nucleosome association [47]. We plotted signal intensity as a function of read length for each sample (S4B Fig) and observed a characteristic profile with a strong peak at <100 bp (nucleosome-free regions) and a long tail of >100 bp fragments (nucleosome-bound regions). Typically, there exist smaller peaks with a periodicity of ~200 bp, corresponding to mononucleosomes, dinucleosomes, etc. In our samples, we observed the initial 200 bp peak but not subsequent peaks, which is likely due to the shallow sequencing depth of our datasets. Moreover, a smaller periodicity of ~10.5 bp was observed (S4B Fig), indicating the helical pitch of DNA as seen in other ATAC-Seq datasets [47]. When reads were partitioned by nucleosome association, we observed a consistent 2:1 (bound:free) ratio amongst all the samples (S4C Fig). Furthermore, the peak read intensity for the nucleosome-free reads in each sample coincided with the transcriptional start site (TSS), which is another characteristic of ATAC-Seq datasets (S4D Fig). Peaks were distributed across the genome for each dataset with promoters and intergenic regions comprising the vast majority of mapped locations (S4E Fig).

Aside from strong correlations amongst samples, we observed similar TSS accessibility profiles for input CM nuclei, Myc- and PCM1-purified CM nuclei, and ENCODE mouse heart DHS-Seq [36] datasets (Fig 5C). While whole-genome analysis established the quality of our ATAC-Seq datasets, we also wished to evaluate their cell type specificity. Therefore, we examined the chromatin accessibility for a marker gene of CMs (Myh6) and fibroblasts (Col3a1). We observed that all three samples showed a high degree of chromatin accessibility at the Myh6 locus, which is consistent with the results of DHS-seq data generated by the ENCODE consortium^36^ for whole heart tissue (Fig 5Di). Cell type specificity of the chromatin accessibility maps was revealed at the Col3a1 locus, which showed relatively little accessibility in all three samples derived from CM nuclei (Fig 5Dii). In contrast, the DHS-seq dataset showed substantial Col3a1 accessibility, which is consistent with the mixed cellularity of the ENCODE tissue samples.

To further assess the biological relevance of the identified ATAC-seq chromatin accessibility peaks for each sample, we performed PANTHER Gene Ontology (GO) analyses for nearest neighbor genes. Consistent with a common cell of origin, all three datasets returned terms containing “cardiac”, “heart”, or “muscle” (Fig 5Ei–5Eiii). Each dataset also returned unique GO terms. Whereas the Nkx2-5 dataset returned terms reflective of active cellular proliferation (Fig 5Eii), the PCM1 dataset returned terms more consistent with cell-cycle exit and terminal differentiation, such as “negative regulation of cell cycle G1/S phase transition” (Fig 5Eiii). Interestingly, the associated GO terms are consistent with prior data showing that PCM1 labels post-mitotic CMs [26] and that a subset of Nkx2-5^+^ CMs retain proliferative activity [48]. Taken together, these studies highlight characteristic features of our ATAC-seq datasets and reveal a remarkable degree of consistency between samples.

### Isolation of podocyte nuclei from adult kidney by PAN-INTACT

**Fig 6:**
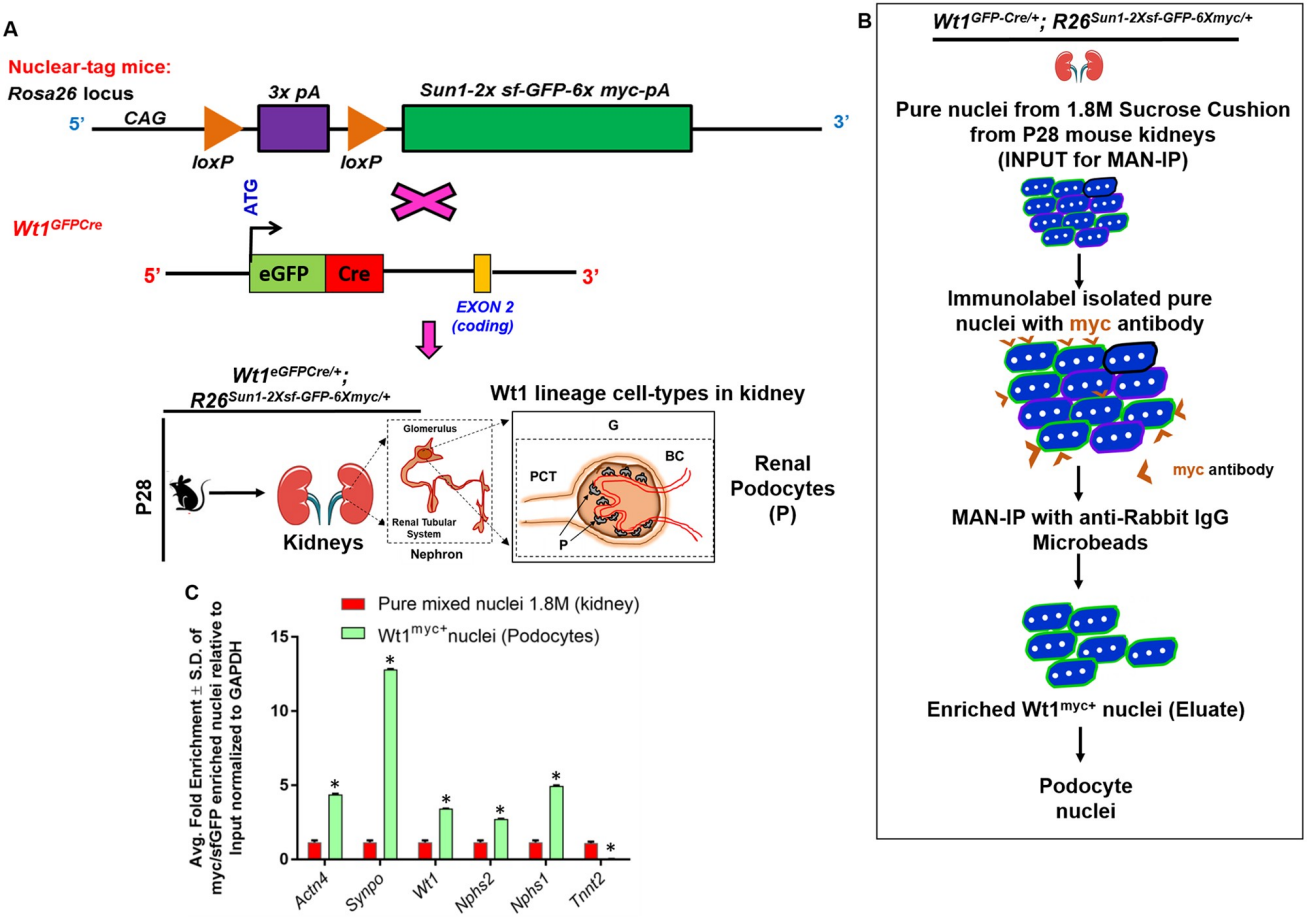
PAN-INTACT enriches for rare cell type specific nuclei from adult mouse kidney. A) R26^Sun1-2xsf-GFP-6xmyc/+^ female mice were bred with Wt1^eGFPCre/+^ males. Upon Cre-mediated recombination of the floxed STOP cassette, the SUN1 fusion protein is expressed on the nuclear membrane in Wt1 lineage positive cells. At P28, Wt1^eGFPCre/+^;R26R^Sun1-2XsfGFP-6-myc/+^ mouse kidneys were harvested. B) Mixed kidney nuclei were purified using a 1.8M sucrose cushion followed by immuno-purification with Myc MAN-IP to isolate podocyte nuclei. C) qRT-PCR analysis demonstrates enrichment of podocyte markers Actn4, Synpo, Nphs2, and Nphs1. The CM marker Tnnt2 was not enriched. 1.8M sucrose-cushion purified nuclei was used as the input for qRT-PCR to calculate fold enrichment, and Gapdh served as an internal standard. Data is represented as average fold enrichment ± S.D. of triplicate reactions for each marker gene over input. Y-axis scale: Linear. * indicates p-value < 0.05 compared with input. Acronyms: Proximal Convoluted Tubule (PCT), Glomerulus (G), Bowman’s Capsule (BC), Renal podocytes (P).

We have so far established that PAN-INTACT successfully purifies lineage-positive nuclei from the heart. However, to increase the generalizability of our approach, we next sought to apply PAN-INTACT for the isolation of cell type specific nuclei from adult kidney, another fibrous tissue source from a highly heterogenous and diverse cellular pool. Therefore, we took advantage of an independent and well-established Cre line (Wt1^GFPCre^) [49] that is known to specifically label podocytes within the kidney. This Cre allele expresses an enhanced green fluorescent protein-Cre recombinase fusion protein (EGFP-Cre) under the control of endogenous Wt1 promoter/enhancer elements. Wt1 is also a key regulator of podocyte function, and reduced Wt1 expression can cause crescentic glomerulonephritis or mesangial sclerosis depending on gene dosage [50]. We bred Wt1^eGFPCre/+^ and R26^Sun1-2xsf-GFP-6xmyc/+^ mice (containing loxP-flanked sequences), such that the resulting offspring (Wt1^GFPCre/+^; R26R^Sun1-2XsfGFP-6-myc/+^) undergo Cre-mediated deletion of the floxed sequences and drive expression of the Sun1-sfGFP-myc-Tag in nuclei of Wt1 lineage positive cells (Wt1^myc+^ nuclei) (Fig 6A).

Although extraction of cardiac nuclei at various ages required extensive optimization (Fig 2C), we were able to rapidly determine the optimal douncing parameters for recovery of nuclei from kidney tissue based on prior observations. While optimizing cardiac nuclei isolation, we noted that the resistance encountered by douncing heart tissue undergoes a rapid decline at the point at which nuclei are optimally isolated. This transition point occurs earlier in younger tissues (less connective tissue). Using this guiding principle, we quickly optimized the number of strokes required for nuclei preparation from P28 kidney by douncing until the resistance dropped, and a smooth suspension was formed. Additional details of the modification used for nuclei isolation from kidney are provided in the Materials and Methods section. To isolate heterogeneous kidney nuclei, we processed P28 kidney tissue and purified the nuclei over a 1.8M sucrose cushion as described earlier (Fig 3) to maintain the cell type heterogeneity of purified kidney nuclei.

Next, we carried out immunoaffinity purification of Wt1^+^ podocyte nuclei using a Myc antibody (Fig 6B). Following elution from the beads, we treated the nuclei with appropriate secondary antibodies and performed confocal microscopy (S5 Fig). This analysis confirmed complete overlap between Myc labeling and sfGFP expression as expected for efficient isolation of podocytes from adult mouse kidney (S5 Fig). Additionally, the calculated sensitivity, specificity, and fold enrichment associated with purification of Wt1^+^ nuclei from multiple independent experiments were highly correlated. To confirm the identity of purified Wt1^+^ nuclei, we isolated nuclear RNA for cDNA synthesis. Then, we performed qRT-PCR analysis using primer sets for specific marker genes. Pure nuclei isolated from the 1.8M sucrose cushion served as the input sample for each enrichment. Wt1^+^ nuclei from the kidney showed increased expression of podocyte markers Wt1, Actn4, Synpo, Nphs2, and Nphs1 (Fig 6C). Consistent with the specificity of the enrichment process, we found that a CM marker was absent from Wt1^+^ podocytes (Fig 6C). Collectively, these data demonstrate successful isolation of cell type specific nuclei from adult kidney tissue.

## Discussion

Here we describe PAN-INTACT, a versatile protocol for isolating cell type specific nuclei from particularly challenging tissues, including the heart and kidneys. We systematically identified favorable douncing parameters to release nuclei from fibrous cardiac tissue. By manipulating the sucrose cushion, we also found that cell type heterogeneity could be preserved. In contrast, we also identified sucrose cushion parameters that enable label-free isolation of cardiac nuclei, which should be particularly useful for application to human tissues. Building on successful extraction of cardiac nuclei, we adapted the mammalian INTACT method to purify cardiac Nkx2-5^+^ nuclei, which express specific markers for CMs, smooth muscle cells, and endothelial cells. Furthermore, we demonstrate that Nkx2-5^+^ nuclei can be used to generate high-resolution chromatin accessibility maps by applying the OMNI-ATAC-Seq protocol. Finally, we show that PAN-INTACT can be adapted to isolate Wt1^+^ podocytes from the kidney of adult mice. PAN-INTACT is an easy, inexpensive, and flexible method for isolating both rare and abundant cell type specific nuclei for a wide variety of transcriptomic and genomic analyses.

The central dogma recognizes the nucleus as a major hub of information transfer within the cell by housing the machinery for DNA replication and RNA transcription. Although translation, which is carried out by ribosomes, represents the final step in the flow of cellular information, the nucleus contains two-thirds of the cell’s information content (i.e. DNA and RNA). Thus, the nucleus can provide a wealth of information about the defining features of a particular cell type. Consequently, cell type specific nuclei isolation by INTACT has been adapted to many model systems, including mammalian brain tissue [22]. Interestingly, isolated mouse and human neuronal nuclei have also been used for single nuclear sequencing (snRNA-seq) [51], which provides the additional advantages afforded by single cell RNA sequencing (scRNA-seq) [52] capabilities. In turn, the insights obtained by performing such detailed transcriptomic and genomic analyses on bulk and single neuronal nuclei have revolutionized our understanding of their lineage specification and function. However, INTACT and snRNA-seq have not been adapted to all tissue types, and a major impediment to broad applicability has been the difficulty of isolating nuclei from fibrous tissues and organs.

In order to enable the utilization of INTACT to isolate adult cardiac nuclei, we used previous protocols as a starting point [22, 25, 28] and systematically optimized each step (Table 1). The ability to easily purify nuclei from a wide variety of tissues paves the way for future studies using the many existing Cre driver lines with exquisite cell type specificity. For example, atrial and ventricular Cre driver lines could be used to profile the transcriptome and chromatin accessibility of individual CM subtypes. Similarly, individual kidney cell types can be examined based on available Cre lines. In addition, since DNA and RNA are collected simultaneously from purified nuclei, numerous additional profiling assays can be performed. ChIP-seq using antibodies for histone marks or transcription factors could provide complementary information in specific cell types, as has been described previously for CMs [25] and neurons [22]. Alternatively, whole genome methyl-cytosine and hydroxymethyl-cytosine sequencing could provide cell type specific epigenetic signatures. Ultimately, further technical improvements could enable snRNA-seq from rare cell types, which would provide nuanced information regarding cell type heterogeneity. To enable such in-depth transcriptional analysis in human tissue, however, newer methods will need to be developed such that genetic tagging of cell type specific nuclei is not required. In this regard, perhaps variable centrifugation or the identification of new cell type specific nuclear markers could make this future goal a reality.

**Table 1:** Systematic optimization of PAN-INTACT.

Parameter	Protocols used in studies referred to in [23] and [25]	Protocol used in study referred to in [17]	This study
**1. Tissue homogenization**	**Bosch 300W Blender followed by Ultra-Turrax mediated homogenization**	**Razor blade mincing**	**Fine-scissor mediated mincing**
**2. Nuclei Extraction**	**8 strokes of dounce with Type A pestle**	**Dounce with Type A pestle followed by 5 strokes of the Type B pestle**	**Variable strokes with Type A pestle (age-dependent optimization as shown in** Fig 2**) followed by 2–3 strokes of Type B pestle**
**3. Nuclei purification**	**2.1 M Sucrose Solution**	**Iodixanol density medium**	**1.8M Sucrose Cushion for purifying mixed nuclei; 2.2M Sucrose Cushion for CM nuclei**
**4. Anti-clumping Reagent**	**None**	**None**	**10% Glycerol**
**5. Antibody for IP**	**αPCM1 (post-mitotic CM only)**	**αGFP (any Cre-recombined Sun-tagged nuclei)**	**αMyc (any Cre-recombined Sun-tagged nuclei)**
**6. Magnetic beads for IP**	**Anti-rabbit IgG Microbeads**	**Protein G Dynabeads**	**Anti-rabbit IgG Microbeads**
**7. Assessing sorting efficiency**	**Flow Cytometry**	**Fluorescence Microscopy and hemocytometer**	**Countess II FL Automated Cell Counter (DAPI light cube); confocal microscopy**
**8. RNA/DNA Purification**	**AllPrep DNA/RNA Mini Kit, Qiagen**	**RNeasy Micro kit (Qiagen), Dneasy Blood and Tissue kit (Qiagen)**	**ZR-Duet DNA/RNA MiniPrep Plus**

Summary of individual INTACT parameters that were optimized and/or modified in this manuscript. Each PAN-INTACT step is shown alongside the corresponding step from previously described protocols for adult heart [25,28] and neurons [22]. The numbers for each parameter in the table correspond to labeled steps in Fig 1.

## Conclusions

Taken together, our data suggest that PAN-INTACT is broadly applicable for profiling the transcriptional and epigenetic landscape of specific cell types. Thus, we envision that our method can be used to systematically probe mechanistic details of cell type-specific functions within individual organs of intact mice.

## Supporting information

S1 Table qRT-PCR primer sequences. (TIF)

S2 Table Buffer composition for lysis buffer. (TIF)

S3 Table Buffer composition for sucrose cushions. (TIF)

S4 Table Buffer composition for nuclei resuspension buffer. (TIF)

S5 Table Nuclei yield for different sucrose gradients across postnatal murine ages. (TIF)

S6 Table Numbers of mapped library reads for each ATAC-Seq sample. (TIF)

S1 Fig Evaluation of beads for antibody-mediated CM nuclei enrichment. A) Fluorescent images showing DAPI staining for (i) CM nuclei and (ii) COS7 nuclei purified from 2.2M sucrose-cushion demonstrating the size difference. (iii) Dynabeads (brown circles) bind to nuclei labeled with PCM1 antibody for 30 minutes. (iv) Upon mixing COS7 nuclei and PCM1-labeled nuclei, Dynabeads M-280 Sheep anti-rabbit IgG beads were unable to specifically bind to immunolabeled nuclei. Red arrow shows a PCM1-labeled nucleus, while green arrows show COS7 nuclei. B) Schematic representation of the MAN-IP steps performed after nuclei purification over a 2.2M sucrose cushion. Nuclei were labeled with an antibody against cardiac nuclear membrane antigen Pericentriolar Material 1 (PCM1) followed by precipitation with anti-Rabbit IgG microbeads. C) Immunofluorescence images showing robust and efficient PCM1 labeling of CM nuclei in the eluate following overnight incubation with PCM1 antibody. The flow through (FT) contains only unlabeled nuclei. Nuclei were counter-stained with DAPI. D) Quantification of four independent experiments yielded estimates of PCM1 MAN-IP of specificity and sensitivity (range in percentage with S.D.) in parentheses. Magnification: 100μm.
(TIF)

S2 Fig Sucrose cushion parameters alter the distribution of heart cell nuclei. qRT-PCR demonstrates heterogeneous cell type nuclei for 1.8M cushion and homogeneous CM nuclei for 2.2M cushion. Specific marker genes, such as Tnnt2 (CM), Wt1 and Upk1b (epicardial), Col1a1 (cardiac fibroblast), and Pecam1 (endothelial) were used in qRT-PCR experiments. Fold enrichment was calculated using cDNA from A) whole heart tissue or B) crude nuclear pellet (not yet purified over sucrose gradient) as a reference. Gapdh served as an internal standard for qPCR. Data is represented as average fold enrichment ± S.D. of triplicate reactions for each marker gene. Y-axis scale: Log2.
(TIF)

S3 Fig Validation of Myc MAN-IP for purifying Nkx2-5 lineage positive nuclei from P1 murine heart. A-C) Confocal images of nuclei in the eluate following Myc MAN-IP on mixed nuclei (1.8M sucrose cushion) extracted from P1 Nkx2-5^Cre/+^; R26^Sun1-2xsf-GFP-6xmyc/+^ mouse hearts. The purified nuclei were stained with antibodies for Myc (A), PCM1 (B), or PLN (C). D-F) Confocal images of nuclei in the eluate following Myc MAN-IP on cardiac nuclei (2.2M sucrose cushion) extracted from P1 Nkx2-5^Cre/+^; R26^Sun1-2xsf-GFP-6xmyc/+^ mouse hearts. The purified nuclei were stained with antibodies for Myc (D), PCM1 (E), or PLN (F).
(TIF)

S4 Fig Comparison of ATAC-seq datasets generated by PAN-INTACT. A) Principle component analysis (PCA) was performed using each biological replicate for the input, PCM1 MAN-IP, and Myc MAN-IP samples. This analysis shows high overall concordance amongst biological replicates and between MAN-IP samples. B) Histograms representing the insert size distribution of sequenced fragments from input, Nkx2-5^+^, and PCM1^+^ ATAC-seq libraries. The average periodicity of insert size distribution from all reads was approximately 200 bp with additional periodicity corresponding to the helical pitch of DNA (~10.5 bp). X-axis represents fragment length in base pairs (bp), and Y-axis represents normalized read density. C) Pie-chart showing genome-wide distribution of nucleosome-bound and nucleosome-free ATAC-Seq peaks. D) Nucleosome-free peaks were plotted for each sample centered on the transcriptional start site (TSS). Peak read density was observed overlying the TSS in each sample. RPKM, Reads Per Kilobase Million. E) The genomic distribution of ATAC-seq reads are depicted as a pie chart for each sample.
(TIF)

S5 Fig Validation of Myc MAN-IP for purification of Wt1 lineage positive nuclei from kidney. At P28, *Wt1*^*eGFPCre/+*^; *R26R*^*Sun1-2XsfGFP-6-myc/+*^ mouse kidneys were harvested, and mixed nuclei were purified over a 1.8M sucrose cushion. Tagged nuclei were isolated by immunoaffinity purification with a Myc antibody, and the nuclei in the eluate were counter-stained with DAPI and visualized by fluorescence confocal microscopy. As expected, all sfGFP^+^ nuclei (green) were also Myc^+^ (red), and the majority of DAPI^+^ nuclei from the 1.8M cushion were both sfGFP^+^ (green) and Myc^+^ (red). Magnification: 100μm.
(TIF)
