# Supplementary figures and images for: PAN-INTACT enables direct isolation of lineage-specific nuclei from fibrous tissues
Source: PLoS One. 2019 Apr 2;14(4):e0214677. doi: 10.1371/journal.pone.0214677 (PMC6445515; doi:10.1371/journal.pone.0214677)

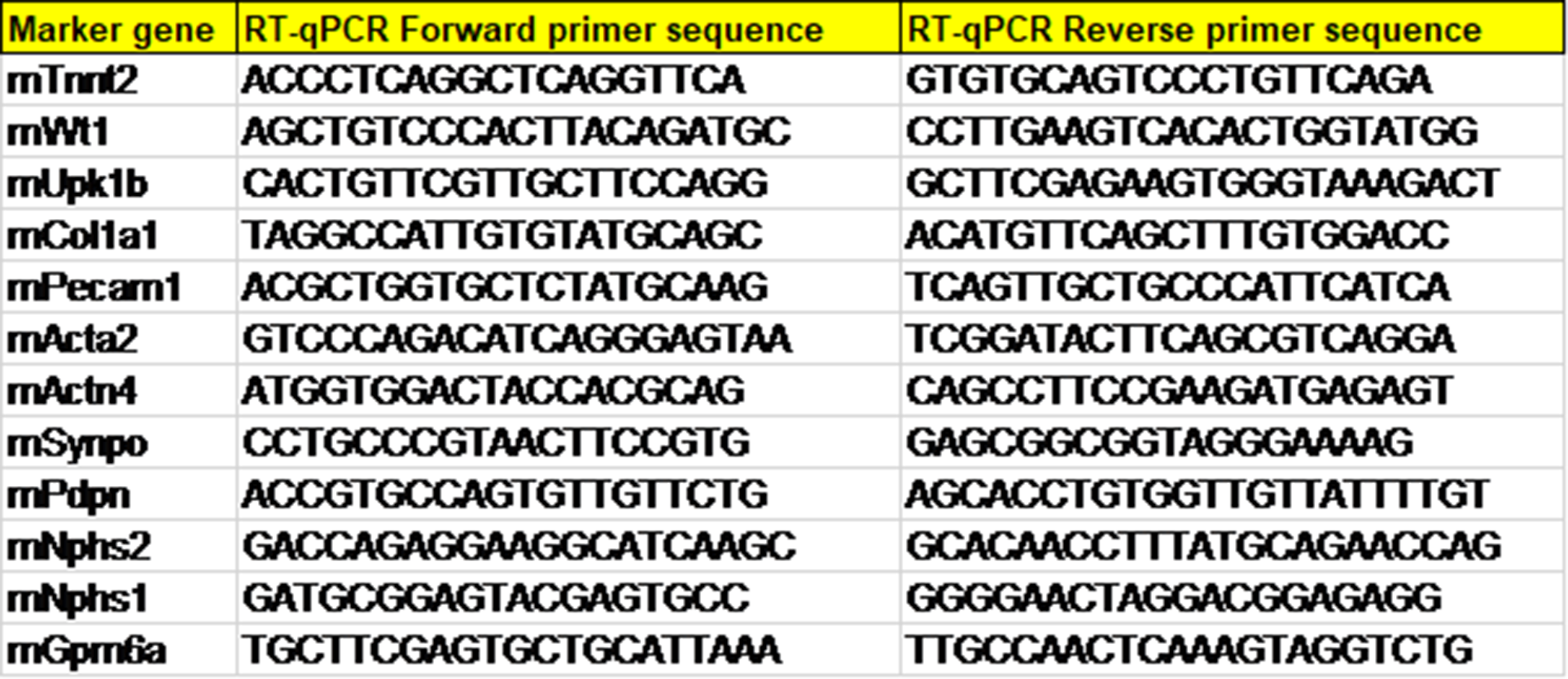

Supplement: S1 Table — (TIF) [file pone.0214677.s001.tif]

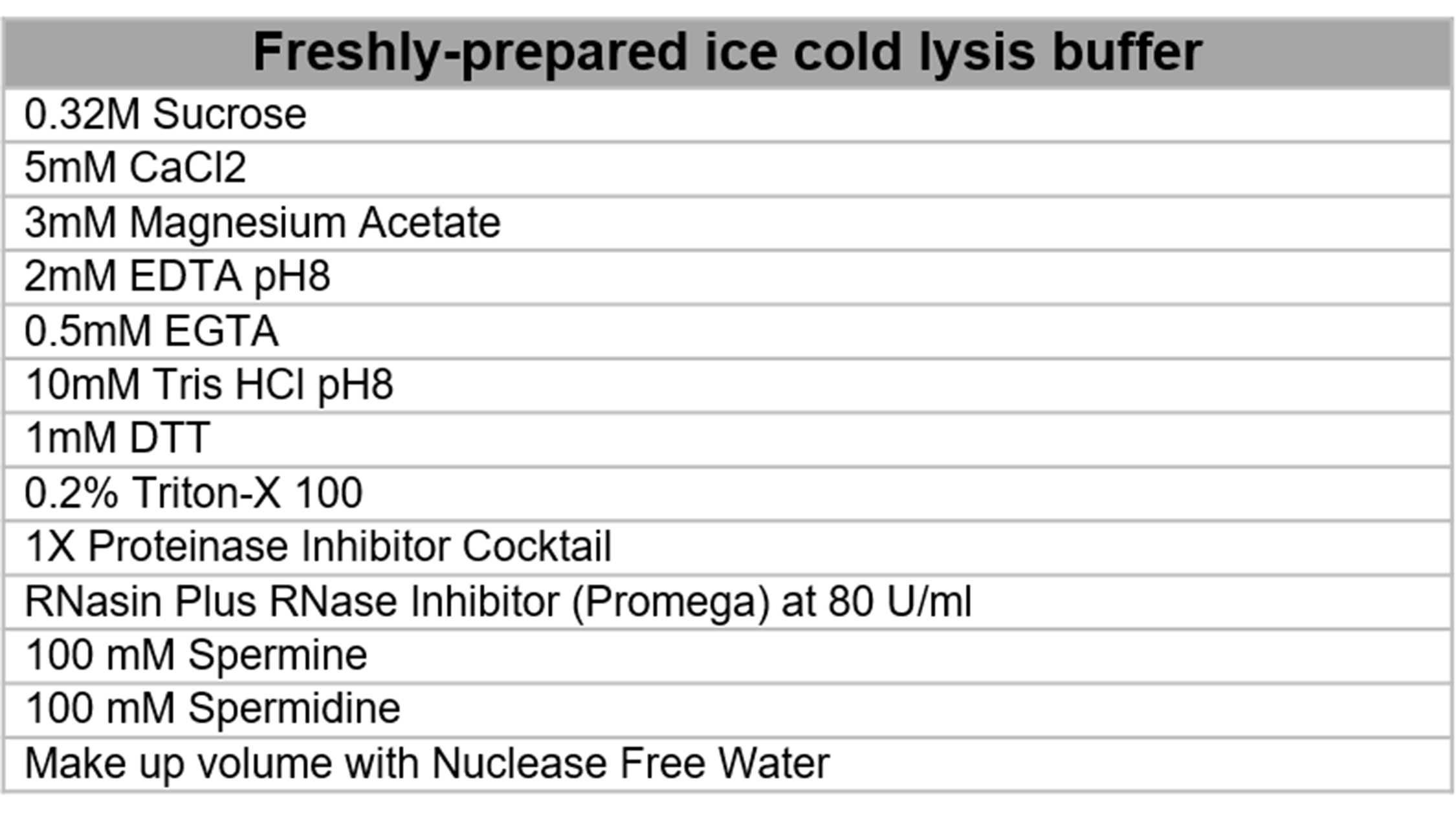

Supplement: S2 Table — (TIF) [file pone.0214677.s002.tif]

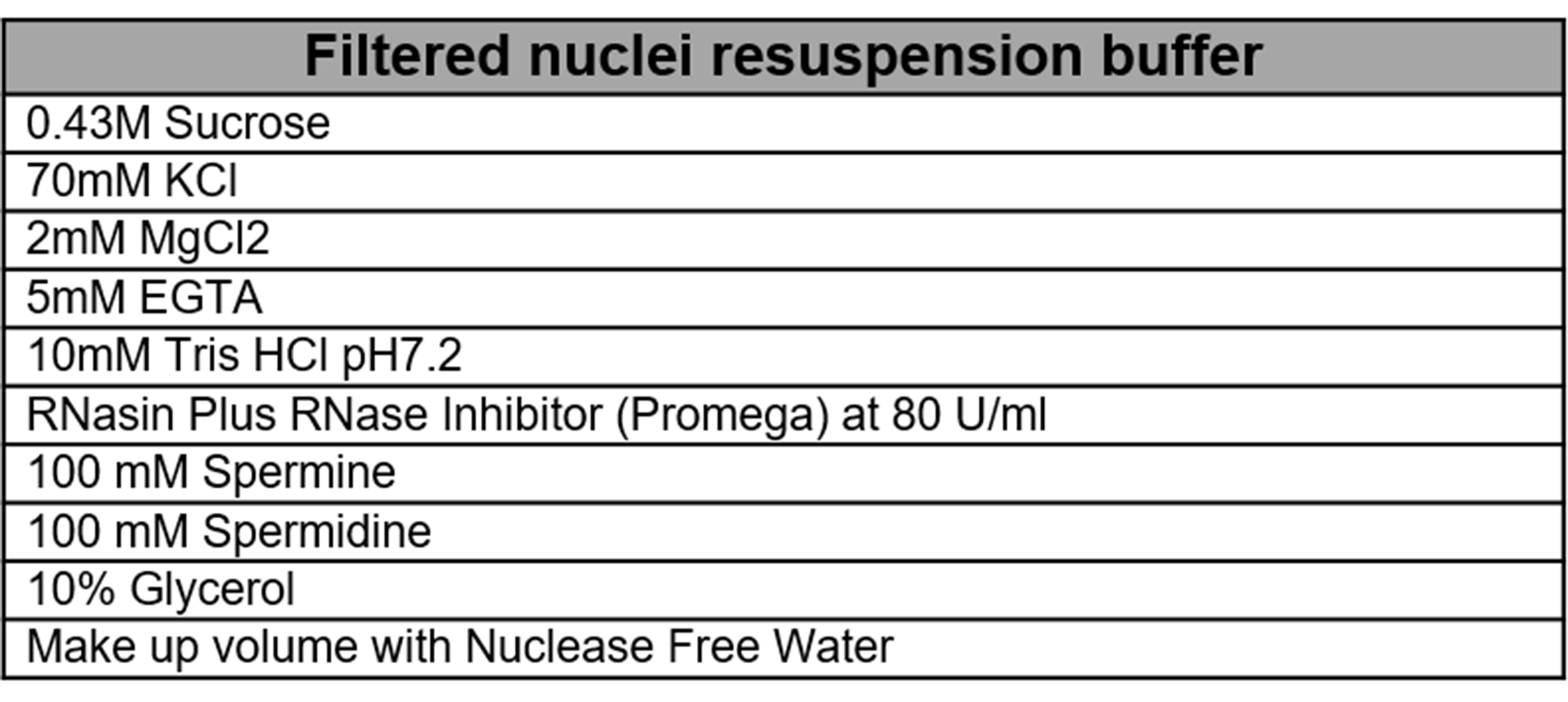

Supplement: S4 Table — (TIF) [file pone.0214677.s004.tif]

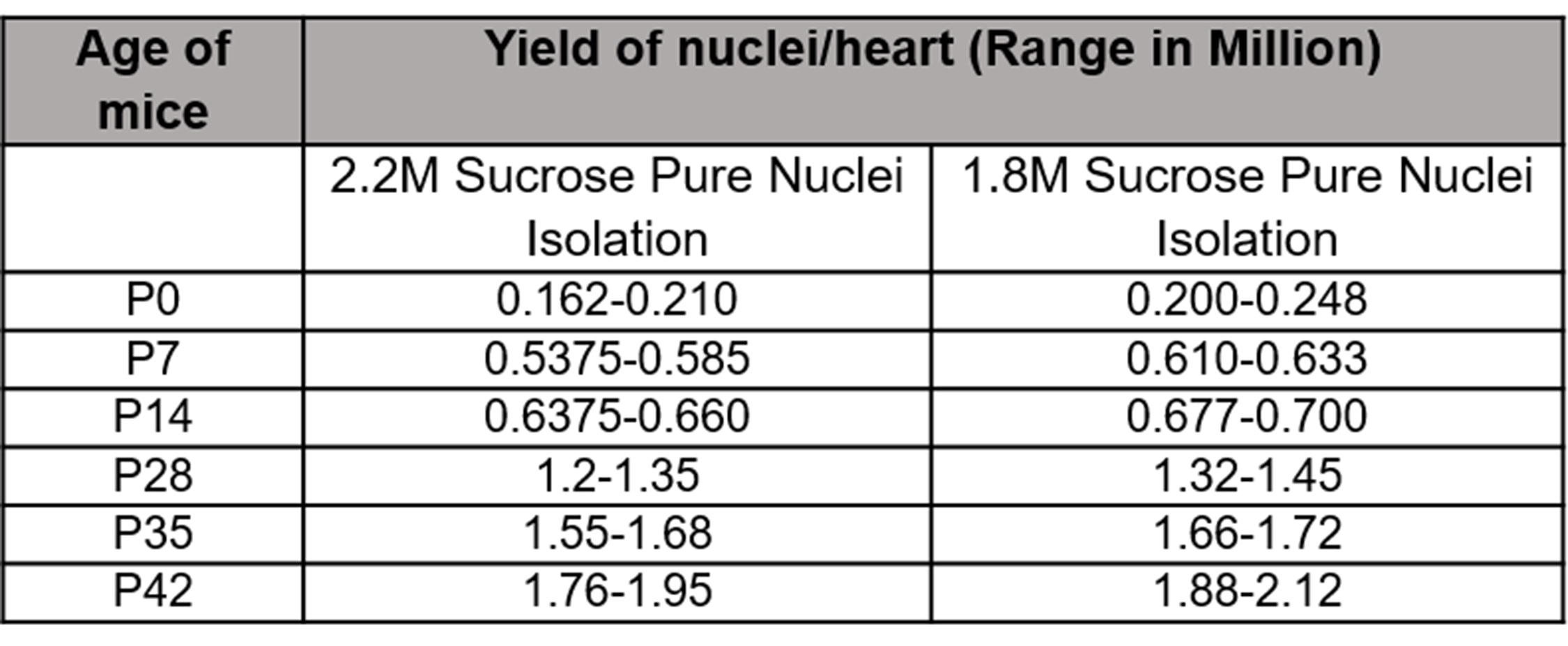

Supplement: S5 Table — (TIF) [file pone.0214677.s005.tif]

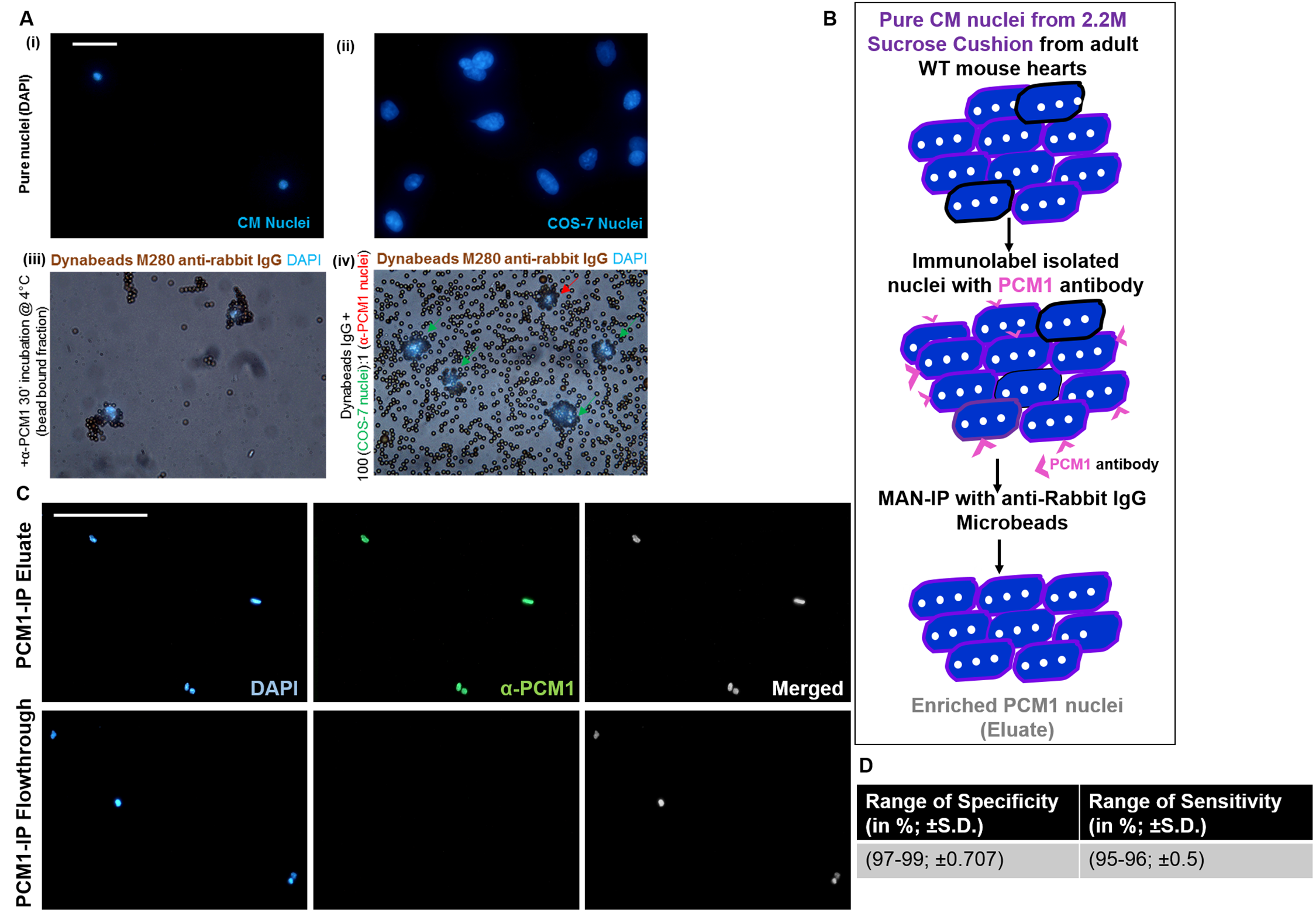

Supplement: S1 Fig — A) Fluorescent images showing DAPI staining for (i) CM nuclei and (ii) COS7 nuclei purified from 2.2M sucrose-cushion demonstrating the size difference. (iii) Dynabeads (brown circles) bind to nuclei labeled with PCM1 antibody for 30 minutes. (iv) Upon mixing COS7 nuclei and PCM1-labeled nuclei, Dynabeads M-280 Sheep anti-rabbit IgG beads were unable to specifically bind to immunolabeled nuclei. Red arrow shows a PCM1-labeled nucleus, while green arrows show COS7 nuclei. B) Schematic representation of the MAN-IP steps performed after nuclei purification over a 2.2M sucrose cushion. Nuclei were labeled with an antibody against cardiac nuclear membrane antigen Pericentriolar Material 1 (PCM1) followed by precipitation with anti-Rabbit IgG microbeads. C) Immunofluorescence images showing robust and efficient PCM1 labeling of CM nuclei in the eluate following overnight incubation with PCM1 antibody. The flow through (FT) contains only unlabeled nuclei. Nuclei were counter-stained with DAPI. D) Quantification of four independent experiments yielded estimates of PCM1 MAN-IP of specificity and sensitivity (range in percentage with S.D.) in parentheses. Magnification: 100μm. (TIF) [file pone.0214677.s007.tif]

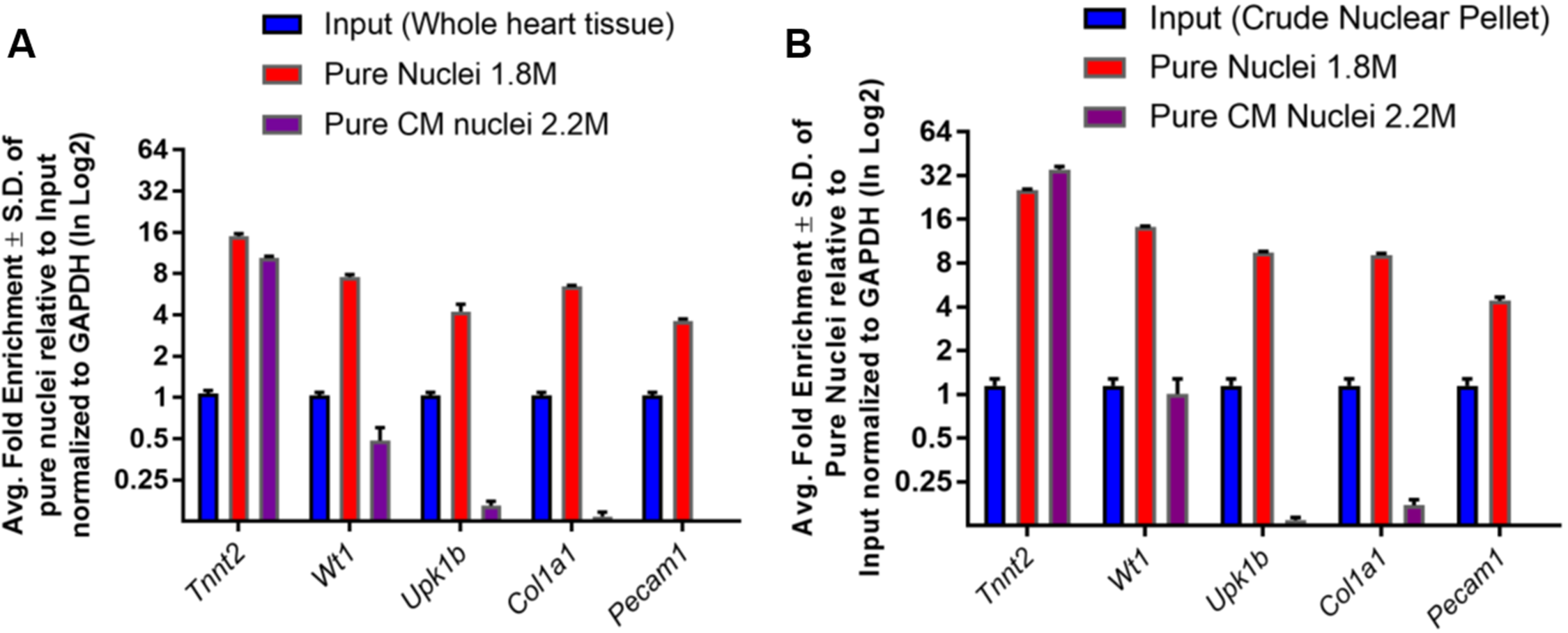

Supplement: S2 Fig — qRT-PCR demonstrates heterogeneous cell type nuclei for 1.8M cushion and homogeneous CM nuclei for 2.2M cushion. Specific marker genes, such as Tnnt2 (CM), Wt1 and Upk1b (epicardial), Col1a1 (cardiac fibroblast), and Pecam1 (endothelial) were used in qRT-PCR experiments. Fold enrichment was calculated using cDNA from A) whole heart tissue or B) crude nuclear pellet (not yet purified over sucrose gradient) as a reference. Gapdh served as an internal standard for qPCR. Data is represented as average fold enrichment ± S.D. of triplicate reactions for each marker gene. Y-axis scale: Log2. (TIF) [file pone.0214677.s008.tif]

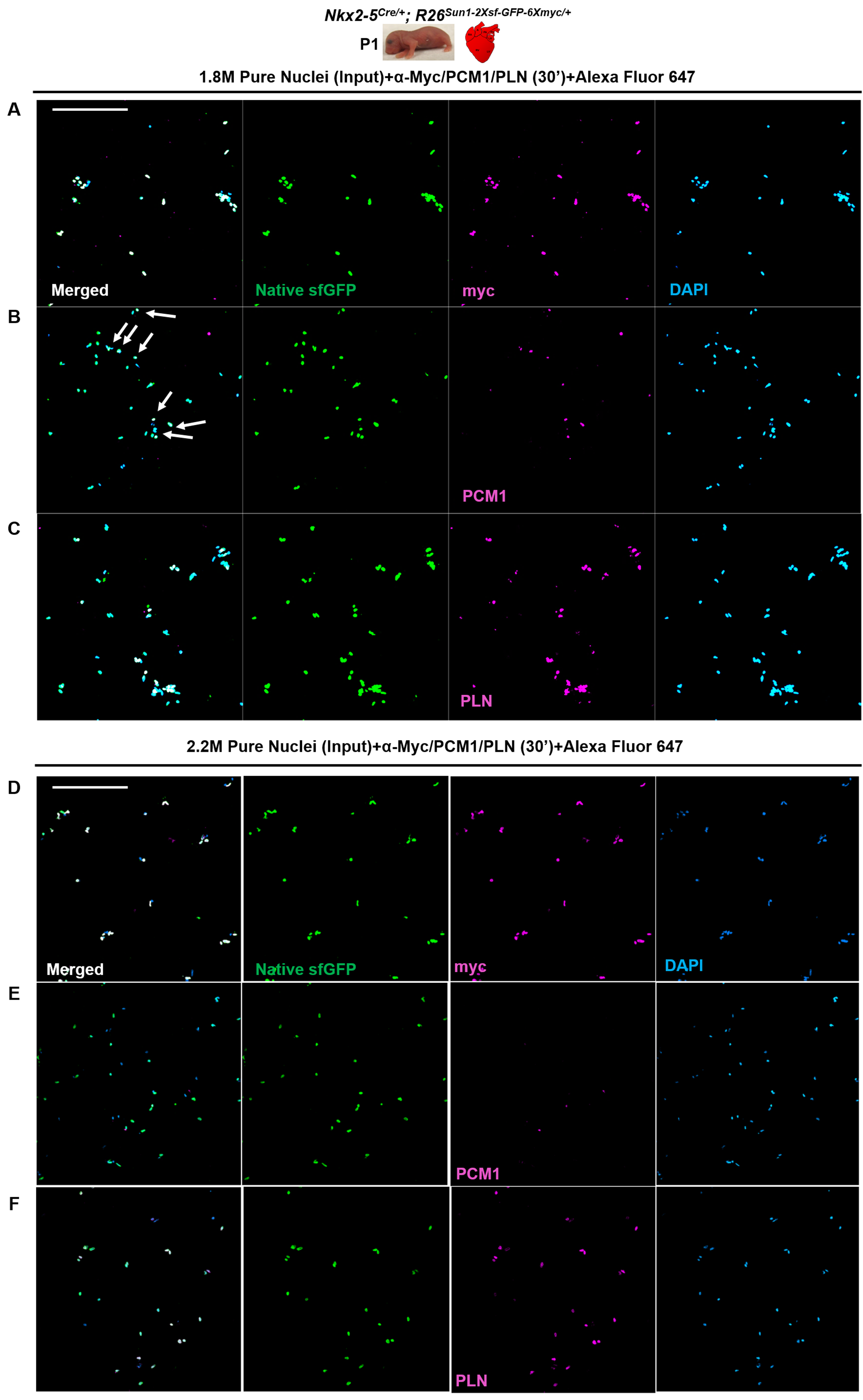

Supplement: S3 Fig — A-C) Confocal images of nuclei in the eluate following Myc MAN-IP on mixed nuclei (1.8M sucrose cushion) extracted from P1 Nkx2-5Cre/+; R26Sun1-2xsf-GFP-6xmyc/+ mouse hearts. The purified nuclei were stained with antibodies for Myc (A), PCM1 (B), or PLN (C). D-F) Confocal images of nuclei in the eluate following Myc MAN-IP on cardiac nuclei (2.2M sucrose cushion) extracted from P1 Nkx2-5Cre/+; R26Sun1-2xsf-GFP-6xmyc/+ mouse hearts. The purified nuclei were stained with antibodies for Myc (D), PCM1 (E), or PLN (F). (TIF) [file pone.0214677.s009.tif]

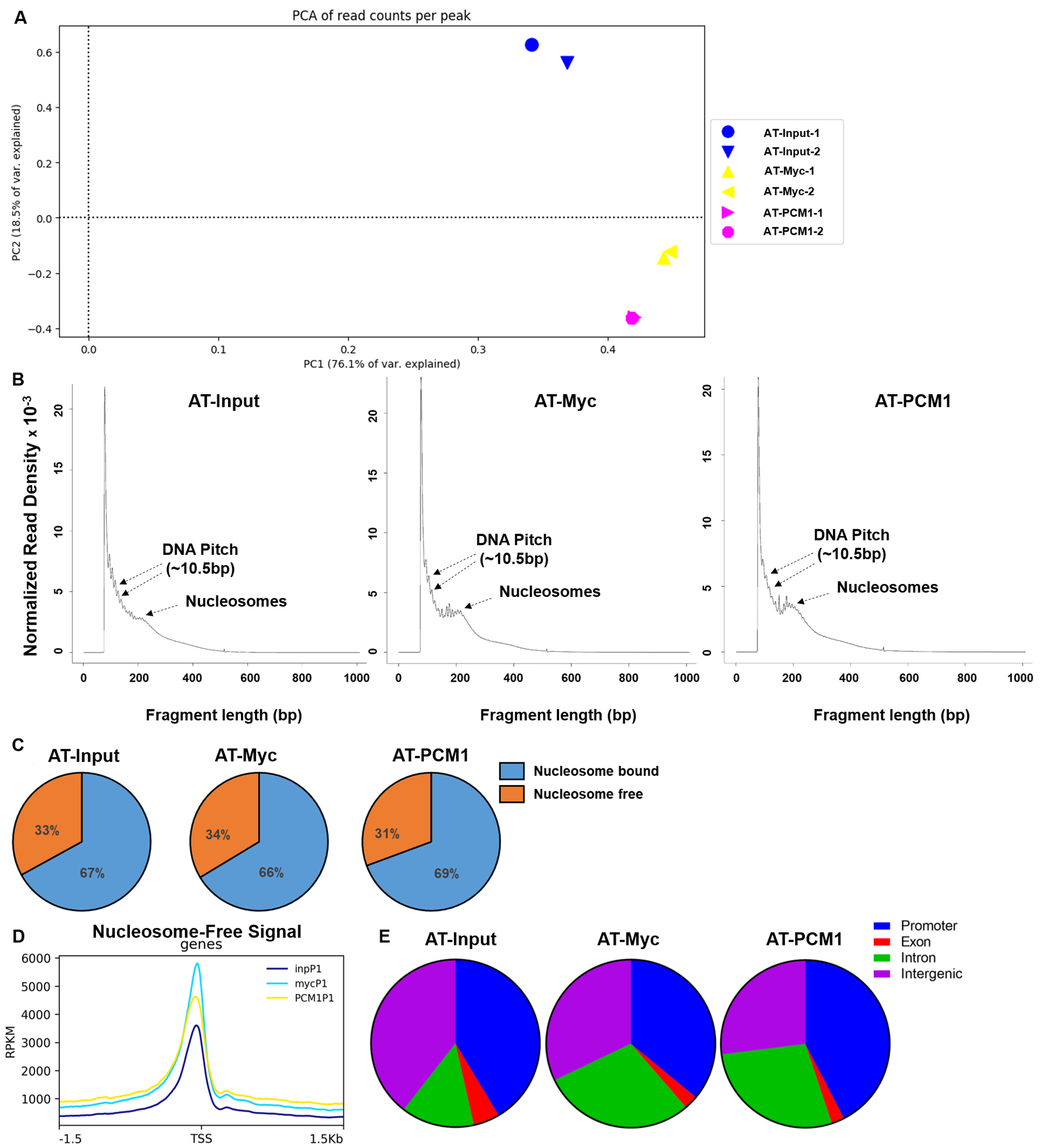

Supplement: S4 Fig — A) Principle component analysis (PCA) was performed using each biological replicate for the input, PCM1 MAN-IP, and Myc MAN-IP samples. This analysis shows high overall concordance amongst biological replicates and between MAN-IP samples. B) Histograms representing the insert size distribution of sequenced fragments from input, Nkx2-5+, and PCM1+ ATAC-seq libraries. The average periodicity of insert size distribution from all reads was approximately 200 bp with additional periodicity corresponding to the helical pitch of DNA (~10.5 bp). X-axis represents fragment length in base pairs (bp), and Y-axis represents normalized read density. C) Pie-chart showing genome-wide distribution of nucleosome-bound and nucleosome-free ATAC-Seq peaks. D) Nucleosome-free peaks were plotted for each sample centered on the transcriptional start site (TSS). Peak read density was observed overlying the TSS in each sample. RPKM, Reads Per Kilobase Million. E) The genomic distribution of ATAC-seq reads are depicted as a pie chart for each sample. (TIF) [file pone.0214677.s010.tif]

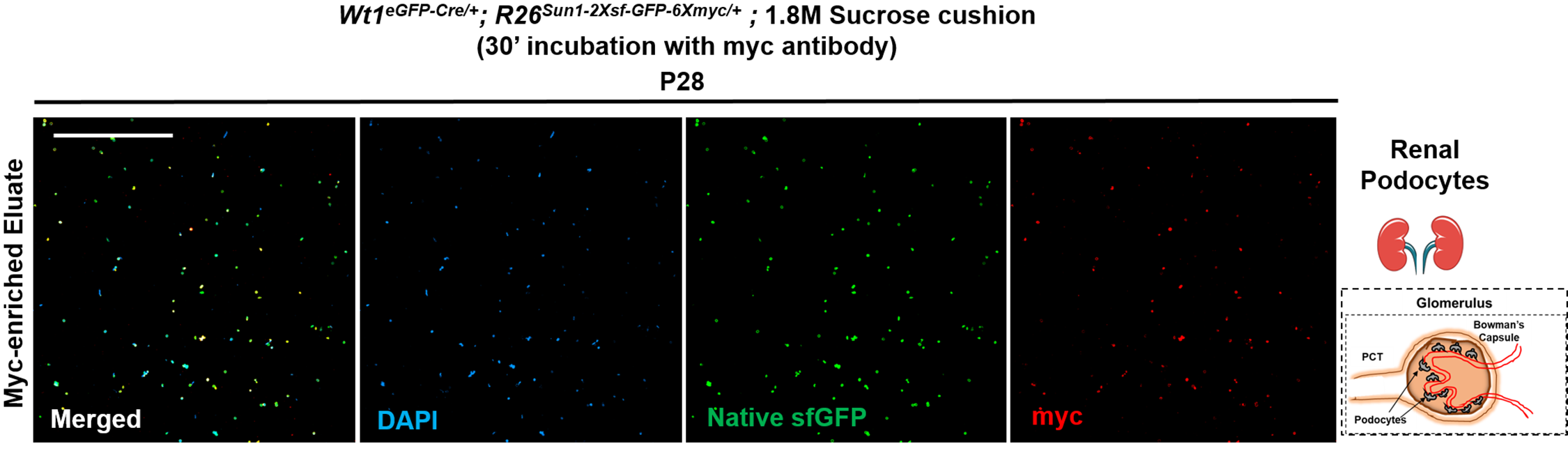

Supplement: S5 Fig — At P28, Wt1eGFPCre/+; R26RSun1-2XsfGFP-6-myc/+ mouse kidneys were harvested, and mixed nuclei were purified over a 1.8M sucrose cushion. Tagged nuclei were isolated by immunoaffinity purification with a Myc antibody, and the nuclei in the eluate were counter-stained with DAPI and visualized by fluorescence confocal microscopy. As expected, all sfGFP+ nuclei (green) were also Myc+ (red), and the majority of DAPI+ nuclei from the 1.8M cushion were both sfGFP+ (green) and Myc+ (red). Magnification: 100μm. (TIF) [file pone.0214677.s011.tif]
